# Applying Principal Component Analysis for Categorized Dimensionality Reduction in DDoS Detection for Software-Defined Networks

**DOI:** 10.12688/f1000research.163778.1

**Published:** 2025-07-28

**Authors:** Keerthana Balaji, Mamatha Balachandra

**Affiliations:** 1 Manipal School of Information Sciences, Manipal Academy of Higher Education, Manipal, Karnataka, India; 2 Manipal Institute of Technology, Manipal Academy of Higher Education, Manipal, Karnataka, India

**Keywords:** DDoS attack, Software Defined Network, CICDDOS2019 dataset, Machine Learning, Feature Engineering

## Abstract

**Background:**

The explosive growth of Software-Defined Networks (SDN) has introduced unmatched scalability with increased flexibility, an essential component of this modern, complicated network infrastructure. While machine learning models promise to be a viable approach for detecting Distributed Denial of Service (DDoS) attacks, their efficiency relies on the quality of the engineered features.

**Methods:**

In this study, an innovative approach for categorizing newly generated features based on domain-specific relevance is applied, followed by Principal Component Analysis (PCA) on each of the categories for dimensionality reduction. These new engineered features represent the originality of the features within the original dataset without losing their integrity by dropping multiple features from the original dataset. These PCA-transformed features, along with other individual features that were not used in the previous step, were merged into a single dataset for further processing using Machine Learning classifiers. This unique methodology not only addresses the curse of dimensionality but also ensures that the meaningful variance within the categories of features is retained. The CICDDoS2019 dataset was used to evaluate the developed model against features engineered from this dataset. The performance of the model was optimized by carefully and strategically selecting features and transforming them into appropriate new features. The algorithm is finely tuned with basic parameters for an effective outcome with reduced run times, and we used the following metrics for evaluation: accuracy, F1-score, precision, recall, and cross-validation to ensure robustness.

**Results:**

This model achieved an overall accuracy score of 0.97, with a dataset of 50,000 values having a multi-class target column with eight different class for categorization. With an expanded dataset having 100,000 values and the same multi-class target column, the model maintained an accuracy of 0.97, proving the reliability and scalability of the model.

**Conclusion:**

This planned and logically structured approach underscores the importance of domain-driven feature generation and categorization.

## 1. Introduction

The transformation of network management through the introduction of Software Defined Networking has advanced the field of networking, and one of its benefits is the separation of the control balance from the data plane. This enables better handling of traffic, provides opportunities for scalability, and improves overall efficiency. Despite these benefits, the centralized nature of Software Defined Networking renders it vulnerable to Distributed Denial of Service (DDoS) attacks. Situations of such attacks require utmost attention as the attacker floods the controller with malicious traffic, with potential targets being the disruption of network operations and the availability of online services. Conventional security approaches often fall short of the growing sophistication of DDoS threats, which highlights the requirement for advanced techniques such as machine learning (ML), which could be an effective solution for detection and mitigation. ML algorithms analyze network traffic patterns to classify malicious behavior; however, their performance depends on the quality of the input features. High-dimensional network traffic datasets contain redundant, noisy, and irrelevant information, necessitating feature selection and engineering to create meaningful features from raw data, such as packet headers, flow durations, and traffic volumes, ultimately improving the accuracy and generalization of models for DDoS attack detection. ^1, 2^ Efficient management of the control plane is performed by the controller, and that of the data plane is performed by switches. ^3^ When attackers send requests with a high bandwidth to fill in the offered bandwidth of the target server, the server becomes inaccessible to authentic users. ^4^

Traditional defense mechanisms include firewalls and general Intrusion Detection Systems (IDS), which struggle to detect some DDoS attacks that have recently become increasingly sophisticated. However, the efficiency of ML depends on the quality of features in the input data. ^5, 6^ The high dimensionality seen in network traffic datasets has many features that indicate packet headers, flow durations, traffic volumes, and other features that describe the nature and volume of traffic passing through networks. Although these features provide vital information related to the network, redundancy, noise, and irrelevant information hinder the detection process. The process of selecting appropriate features and engineering new meaningful features from available features is vital in such models. ^7– 9^ Raw features include the data obtained from the traffic from the basis for the input features used in ML models. ^10, 11^ Such features play a key role in improving the capacity of a model to detect attacks against systems and networks, and they are also good at generalization. ^7, 8^

Machine learning is capable of quickly classifying attack traffic from benign traffic (binary), but the complicated forms of DDoS attacks owing to advanced evasive techniques with multiple types of traffic combined in a single attack can be challenging. ^12^ To combat such complicated attacks, researchers have used Computational Neural Networks (CNN), a deep learning model, and have considered Recurrent Neural Networks (RNN) that are efficient in traffic classification. ^13, 14^ Effective principles such as Principal Component Analysis (PCA) have proven to be very effective in addressing many issues, particularly the curse of dimensionality, by transforming higher-dimensional data into lower-dimensional data.

The model created in this study was evaluated using the CICDDoS2019 dataset, which is publicly available and used by many DDoS detection studies. Data preprocessing was performed according to the proposed methodology and used to train ML models, such as Gradient Boosting Machines, Random Forest, and Neural Networks. The performance was compared against globally transformed features using PCA, with results demonstrating significant improvements in the accuracy and robustness of the simple ML model.

The work done with the proposed innovative technique for feature engineering and classification using a simple ML model is presented in the following sections. Section 2 focuses on the literature related to DDoS detection and dimensionality reduction along with the techniques used for the same. Section 3 outlines the proposed methodology, which includes categorization of features and PCA-based dimensionality reduction. Section 4 explains the setup and results of the experiments, along with a discussion of the results. Section 5 summarizes the output of the experiment to conclude and extends the research directions for future work.

## 2. Related work

Abdulhammed et al. ^15^ explored the improvement of IDS using ML. The focus was primarily on reducing the dimensionality of the CICDDoS2017 dataset using autoencoders and PCA to enhance the performance of the classifier. Classifiers, such as Random Forest, Linear Discriminant Analysis, and Quadratic Discriminant Analysis, were tested. This was followed by a new performance metric, CombinedMc, to evaluate the classification of multiple classes better. This study achieved an accuracy of 99.6%, along with other key metrics, by significantly reducing the feature dimensions. Distribution-based balancing was used in this study to address the imbalance between classes.

Riydh et al. ^16^ used a proactive feature selection model with a nature-inspired optimization algorithm was used by Riydh et al., ^16^ for the selection of relevant features in the CICDDoS2019 dataset. Algorithms such as Random Forest (RF), K-Nearest Neighbour (KNN), and Support Vector Machine (SVM) were implemented for the classification of normal traffic from malicious traffic. This model is shown to outperform existing methods in terms of the detection rate, overall accuracy, and reduction in false positives. This shows the importance of the feature engineering step for machine learning processing and its contribution to the performance of ML algorithms.

Another study highlighting the importance of feature engineering was conducted by Pegah et al., ^17^ wherein the proposed model worked on an Ensemble Feature Selection method with a multi-aspect perspective. The relevant features are selected based on each type of attack, along with a combination of statistical filtering techniques and machine learning algorithms. Prediction times can be reduced by focusing on key features in the dataset, which increases the performance of the ML algorithm. This, in turn, improves the mitigation capabilities of the algorithm. A more informative and precise representation of the traffic data adds to the overall performance of the algorithms.

In a review by Muhammed et al., ^18^ the importance of ML power for the detection of DDoS attacks was summarized based on references from numerous studies. This summary highlights the significant impact of the appropriately chosen dataset and the features selected from the dataset. This step helps researchers and practitioners develop robust solutions for handling DDoS attacks.

A deep learning-based approach that leverages the advantages of RNN and LSTM ML models was developed by Jiyeon *et al.* ^19^ The N-BaIoT dataset was used in this study, which simulates botnet attacks on multiple IoT devices. Approximately 115 features presented within the dataset were categorized into five groups, and the primary key was based on the time window, which provided the best performance.

In another study by Muhammad *et al.* ^20^ conducted as a systematic review, the detection of DDoS attacks was focused on backward elimination, chi-squared tests, and information gain scores for the creation of datasets with significant features. This optimization was shown to increase the efficiency of the tested machine learning models, which were fine-tuned and tested. A feature reduction of up to 68% was achieved, with a minimal accuracy loss of 003%. This strategic combination of feature and machine learning was validated using cross-validation and AUC analyses to mitigate overfitting and collinearity. Among the various algorithms, K-nearest neighbors (KNN) performed the best overall, followed by SVM. Random Forest (RF) performs well on low-dimensional datasets with discrete features, although it is simple and quicker than the others.

Various studies have chosen the best features to improve the performance of the chosen ML model. The important works related to feature engineering used in DDoS attack detection is summarized in Table 1. Most of them have binary classification of attacks and benign traffic. Some studies in the literature have proposed models that perform better with both binary and multi-class classification, but their performance is less than that of binary classification. Based on the literature and data compilation from multiple studies, the following research gaps were identified:

**Table 1.** Important works highlighting feature engineering in DDoS detection.

S.No.	Dataset used	Feature engineering	Feature reduction	Technique used	Class labelling	Accuracy	Reference
1.	IoT-CIDDS	Yes	Yes	5 different ML algorithms	Single	-	^7^
2.	CICDDoS2019	No	Yes	CNN/BiLSTM	Binary	94.52%	^29^
3.	CICDDoS2019	No	Yes	RF, DT, ADA, XGB, MLP, DNN	Binary	99.97%	^30^
4.	CICDDoS2019	No	Yes	GB, ADA, CB	Binary Multi	99.3% 97%	^31^
5.	CICDDoS2019	No	Yes	RF	Binary	99.99%	^32^
6.	CICIDS2019 and CICIDS2017	No	Yes	DT, MLP, XGB, RF	Binary Multi	99.2% 98.83%	^33^
7.	CICIDS 2017 and CICDDoS 2019	No	Yes	RF *, GB, Weighted Voting Ensemble (WVE), KNN, LR	Binary	99.0%	^4^
8.	CICDDoS2019	No	Yes	RF *, LGB, CatBoost, CNN	Binary	99.9%	^34^
9.	CICDDoS2019	No	Yes	RF, ANN, KNN, BNN	Binary	99.7%	^35^
10.	CICDDoS2019 KDD-CUP1999	No	Yes	CNN *, SGD, DT, RF	Binary	98.0%	^3^
11.	CICDDoS2019	Yes	Yes	RF	Multi	97.0%	-

* Best performing algorithm in each study.

## 3. Research gaps

1. The implemented feature selection techniques drop certain features that could possibly misrepresent the importance of the class they represent.
2. Binary classification using such selected features has a limited ability to classify modern multi-class types of complicated attacks.
3. The importance of engineering new features from existing ones could improve the representation of data for the efficient classification of various types of traffic.

## 4. Setup used for the experiment

**Figure 1.**
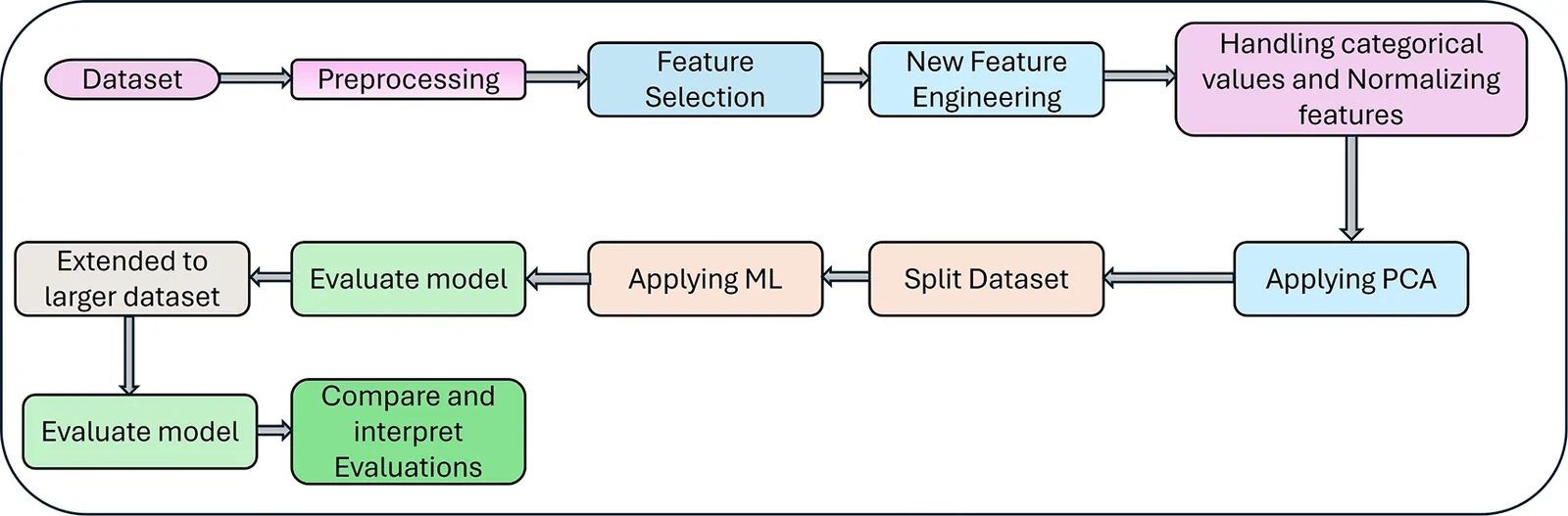
Shows the flow of work done in this study.

Here, the details of the dataset used, the new features engineered, the features dropped, and the detection algorithm used are described. In Figure 1, the steps used in this study, including feature selection, feature engineering, splitting the dataset, and the rest of the steps, are summarized.

### 4.1 Dataset description

The CICDDoS2019 dataset used in this study was published by Sharafaldin *et al.,* ^21^ which is a good fit for testing models developed to detect DDoS attacks in SDNs *.* In this dataset, created using actual traffic, there are more than 80 features, which form a good benchmark dataset for use in DDoS attack detection studies. There are multiple types of attacks that utilize TCP/UDP protocols. Compared to older datasets, this dataset sets a benchmark that includes 12 different DDoS attack types, with categories as reflection- or exploitation-based.

### 4.2 Data preprocessing

Before training the model, preprocessing of the dataset is an essential step for removing noise and reducing redundant and unnecessary data. This step is crucial and helps increase the efficiency of the model performance by reducing the complexity caused by the features within the dataset.

4.2.1 Handling missing and null values

Missing and null values affect the accuracy and precision of the model’s efficiency. In this study, the missing values were replaced as null values and all the null values were then imputed with ‘0,’ to maintain uniformity which improves the performance of the model.

4.2.2 Feature selection and engineering

In our study, this was the most important step, as the design aimed to identify the best features contributing to the classification task. New features are generated by combining as many features as possible and passing them to the model without compromising its efficiency. The following equations show the combinations of features for generating new features:

In this step, 46 features were combined logically and reduced to 16 new features. This reduction of 30 feature sets (46 original features – 16 new features generated = 30 features reduced overall) considerably reduced the complexity of computation of the model and contributed to the performance of the model. After generating these new features, the original features used in the creation of these new features were dropped before further processing the dataset.

**Importance of the new features generated:**

1. **Total Length Packets:** The total data transferred in a flow is calculated by combining the total length of the forward and backward packets.
2. **Avg Packet Size:** The average packet size was calculated by dividing the total length of packets by the total number of packets.
3. **Total Packets:** This represents the total number of packets in a flow by summing the total forward and backward packets.
4. **Total Bytes:** This gives the total number of bytes transferred in a flow by summing the total length of forward and backward packets.
5. **Average Packet Length:** The average packet length was calculated by averaging the mean packet length, mean length of the forward packet, and backward packets.
6. **Flow IAT Aggregate:** The various inter-arrival time statistics flow IAT mean, std, max, and min were used to provide a comprehensive overview of the timing patterns in the flow.
7. **Fwd IAT Aggregate:** By combining various forward inter-arrival time statistics, the forward IAT mean, std, max, and min provide a comprehensive overview of the timing patterns in the forward traffic.
8. **Bwd IAT Aggregate:** Here various backward inter-arrival time statistics, backward IAT mean, std, max, and min, are combined to provide a comprehensive overview of the timing patterns in the backward traffic.
9. **Total Header Length:** The total header length of packets in a flow was calculated by summing the forward and backward header lengths.
10. **Total Segment Size:** This feature calculates the total segment size of packets in a flow by summing the average forward and backward segment sizes.
11. **Subflow of Total Packets:** This feature calculates the total number of packets in the subflows by summing the forward and backward subflows.
12. **Subflow Total Bytes:** This feature represents the total number of bytes in the subflows by summing the forward and backward subflow bytes.
13. **Flag Aggregate:** This feature combines various flag counts and SYN, RST, ACK, URG, and CWE Flags to provide information about the type of traffic and potential attacks.
14. **Flow Speed Ratio:** This feature calculates the ratio of bytes per second to packets per second, providing insights into the efficiency of the flow. It uses flow bytes and packet features.
15. **Active Time Aggregate:** This feature combines various active time statistics, active mean, std, active, and min, to provide insights into the periods of active data transfer.
16. **Idle Time Aggregate:** This feature combines various idle time statistics Idle mean, std, max, and min to provide insights into the periods of inactivity in the flow.

4.2.3 Handling categorical values

The Label column describes the type of attack and its multiple labels, which are designated as target columns. These values were converted to numerical values using the LabelEncoder from the Scikit library. Each of these values was assigned a numerical value designating an individual category, and then processed further through the model.

4.2.4 Normalizing features for improved model performance

Normalizing Features is vital in ML, as it ensures that the values of the various features are scaled for equal contributions to the process of learning the model. We employed StandardScaler to mitigate the influence of large-scale features and to inhibit their domination during the decision-making step. Its capability to make decisions by considering the maximum possible complex patterns and improving predictive accuracy is enhanced in this step.

### 4.3 Importance of applying PCA to the dataset

This was followed by processing these categorical features using Principal Component Analysis (PCA) and passing these values into a machine learning algorithm for classification into multi-class classifications. This study consists of two major parts: feature categorization and basic model evaluation for classification.

### 4.4 Splitting of data

Another important step is splitting the data into training and testing sets. This was performed with 70% of the data used for training, and 30% of the data were used for testing. The preprocessed CICDDoS2019 dataset obtained in the previous step was passed through for splitting. The train_test_split package from the Sklearn library was used for this task.

### 4.5 Application of the algorithm

The RF model was used in this study for this classification task using the libraries for this model. The parameters were set with the number of trees at 100 and the maximum depth at 2. The training part of the data was used to learn patterns and differentiate multiple traffic types. This model was evaluated using the unseen part of the dataset and accuracy scores. This important step, along with its parameters, served as the foundation for further analysis and improvement of the model.

### 4.6 Assessment of model performance

The performance of the model was evaluated using the following evaluation metrics: F1 score, Accuracy, Precision, Recall, ROC-AUC score, confusion matrix, and log loss function. A higher ROC-AUC score indicates a better ability to discriminate between multiple classes. The log loss is another important value that indicates the uncertainty of the model in its predictions, where lower values indicate a higher confidence in the predictions. Finally, the confusion matrix provides details of the predictions made by the model and shows the values for each class, allowing us to analyze the specific error types (false positives and negatives). Overall, these metrics provide a better understanding of its advantages and disadvantages, locating potential areas for improvement in making informed decisions.

## 5. Results and discussion

In this study, the CCIDDoS2019 dataset was used after processing it for feature engineering, where the important features were retained and new features were generated. Two datasets with 50,000 and 100,000 rows were created with multiple classes in the target column. This dataset was processed using a Random Forest classifier, with the hyperparameters kept constant throughout both types of iterations. The datasets were split into training and test sets and then passed through the model, and evaluation metrics were performed on this output. The new features generated considerably improved the classification performance of the model and showed consistency through the evaluation metrics. The values from PCA on the features generated and using these PCA values as part of the dataset considerably reduced the dimensions but retained the interaction and contributions of the features within the dataset. This step reduces the running time of the model with reduced dimensions, resulting in less processing time for the model while retaining the importance of the features.

Observations from this study include feature engineering as a key component and critical component in the enrichment of the dataset. The features derived, such as total packets, Flow IAT aggregate, Subflow Total Packets, and Flag aggregate, encapsulate domain-specific knowledge. This concept increases the representational power of the dataset and the ranking of these features from both the datasets used is presented as a Table ( Table 2). The importance of feature engineering, which can retain the representation of the original dataset, has been given considerable importance in previous studies on machine learning for DDoS attacks. ^22– 24^ Additional aggregated metrics, such as Active Time Aggregate, Idle Time Aggregate, enabled the model to effectively capture temporal and flow-based behavioral patterns within network traffic.

**Table 2.** Features – Importance by ranking in the model.

50k dataset Feature: Importances	100k dataset Feature: Importances
• packet_characteristics_PC2: 0.1523 • traffic_metrics_PC1: 0.1436 • traffic_metrics_PC3: 0.1231 • time_features_PC1: 0.1121 • packet_characteristics_PC1: 0.0952 • traffic_metrics_PC2: 0.0653 • time_features_PC4: 0.0567 • header_flags_PC1: 0.0564 • packet_characteristics_PC4: 0.0499 • traffic_metrics_PC6: 0.0327	• time_features_PC1: 0.1444 • packet_characteristics_PC2: 0.1310 • traffic_metrics_PC3: 0.1299 • traffic_metrics_PC1: 0.1174 • packet_characteristics_PC1: 0.0865 • time_features_PC4: 0.0816 • traffic_metrics_PC4: 0.0724 • header_flags_PC1: 0.0581 • packet_characteristics_PC4: 0.0490 • packet_characteristics_PC3: 0.0355

Running PCA on each of the categories separately ensured that the high-dimensional space within the features was transformed into a compact representation without any loss of information. Retaining a variance of 95% was a significant achievement for each category, and highlighted the importance of most features with reduced noise and redundant dimensions. Retaining the importance of features and reducing noise are essential components of ML algorithms. These algorithms are sensitive to noise and reducing them to the maximum possible extent is an important step in these models. ^25, 26^

**Figure 2.**
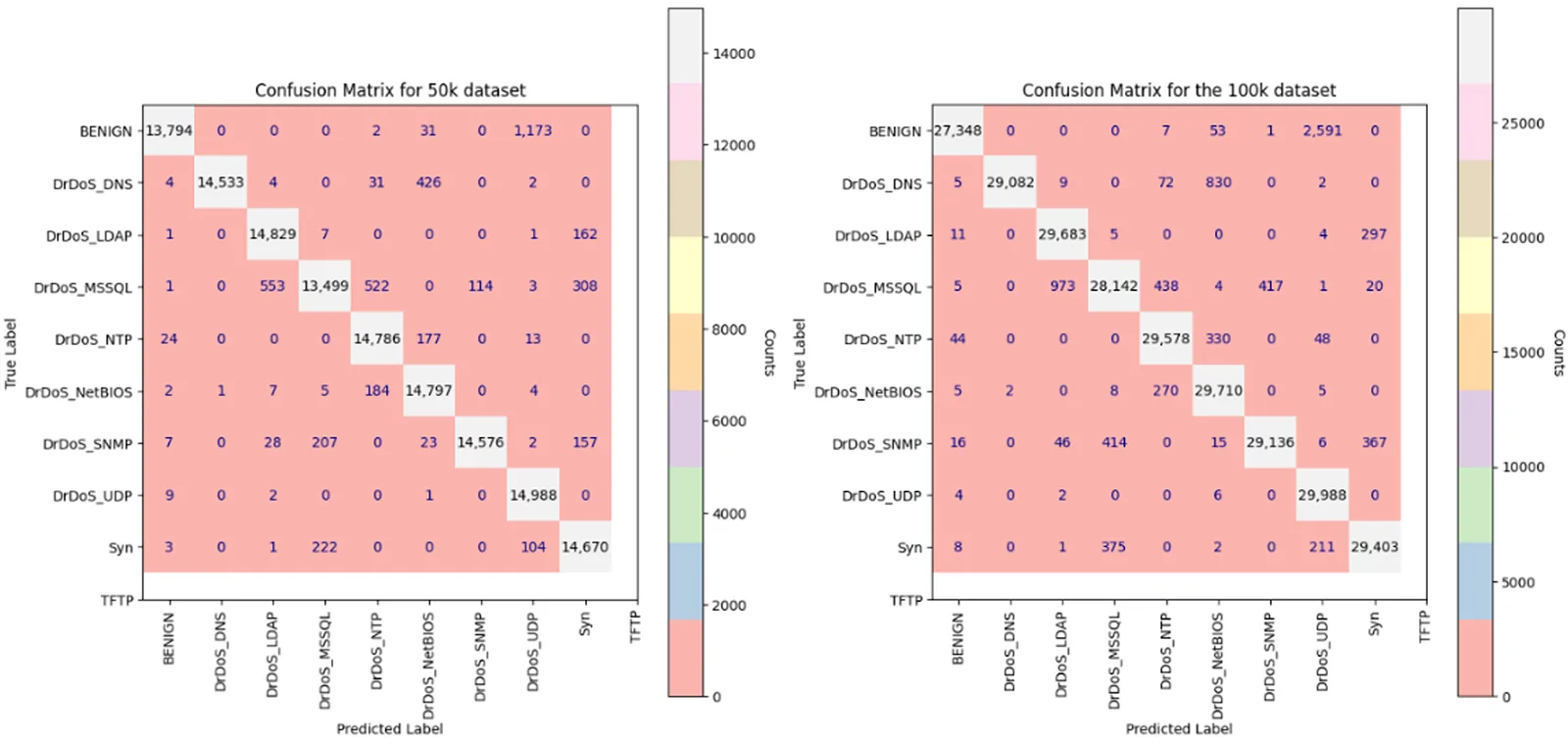
Confusion matrix from the output of running the two different datasets (50k and 100k) using the Random Forest classifier.

In machine learning models, performance against multi-class target sets is an essential feature, depending on the field of interest. ^27, 28^ In areas such as DDoS detection, the capability of the model plays a major role in real-world scenarios. Our model achieved the highest possible accuracy in classifying the various classes within the target column, and this could primarily be a result of the retention of importance within the dataset, although the dimensions were reduced categorically using PCA. This capability is evident from the distribution observed within the confusion matrix, where the false positives are minimal compared to the large size of the datasets used in this study. The confusion matrix for both the datasets (50k and 100k) is shown in Figure 2. This novel concept of categorized PCA successfully retained the importance of the dataset values. The stratified train-test split handled the bias in the evaluation and ensured the class proportions within the training and testing datasets, mirroring the distribution in the original dataset ( Table 3).

**Table 3.** Table showing the evaluation metrics for each of the classes within the dataset.

50k dataset evaluation metrics					100k dataset evaluation metrics				
	precision	recall	f1-score	support		precision	recall	f1-score	support
0	1.00	0.92	0.96	15000	0	1.00	0.91	0.95	30000
1	1.00	0.97	0.98	15000	1	1.00	0.97	0.98	30000
2	0.96	0.99	0.97	15000	2	0.97	0.99	0.98	30000
3	0.97	0.90	0.93	15000	3	0.97	0.94	0.95	30000
4	0.95	0.99	0.97	15000	4	0.97	0.99	0.98	30000
5	0.96	0.99	0.97	15000	5	0.96	0.99	0.97	30000
6	0.99	0.97	0.98	15000	6	0.99	0.97	0.98	30000
7	0.92	1.00	0.96	15000	7	0.91	1.00	0.95	30000
8	0.96	0.98	0.97	15000	8	0.98	0.98	0.98	30000
Accuracy			0.97	135000	Accuracy			0.97	270000
Macro avg	0.97	0.97	0.97	135000	Macro avg	0.97	0.97	0.97	270000
Weighted avg	0.97	0.97	0.97	135000	Weighted avg	0.97	0.97	0.97	270000
Cross-validated F1-scores: [0.9599662 0.99972225 0.99980002 0.99974445 0.9549608] Mean F1-score: 0.9828387424457539					Cross-validated F1-scores: [0.99334321 0.99971667 0.99975556 0.99977223 0.95895865] Mean F1-score: 0.9903092619288781				

Class mapping: [‘BENIGN’: 0, ‘DrDoS_DNS’: 1, ‘DrDoS_LDAP’: 2, ‘DrDoS_MSSQL’: 3, ‘DrDoS_NTP’: 4, ‘DrDoS_NetBIOS’: 5, ‘DrDoS_UDP’: 6, ‘Syn’: 7, ‘TFTP’: 8].

**Figure 3.**
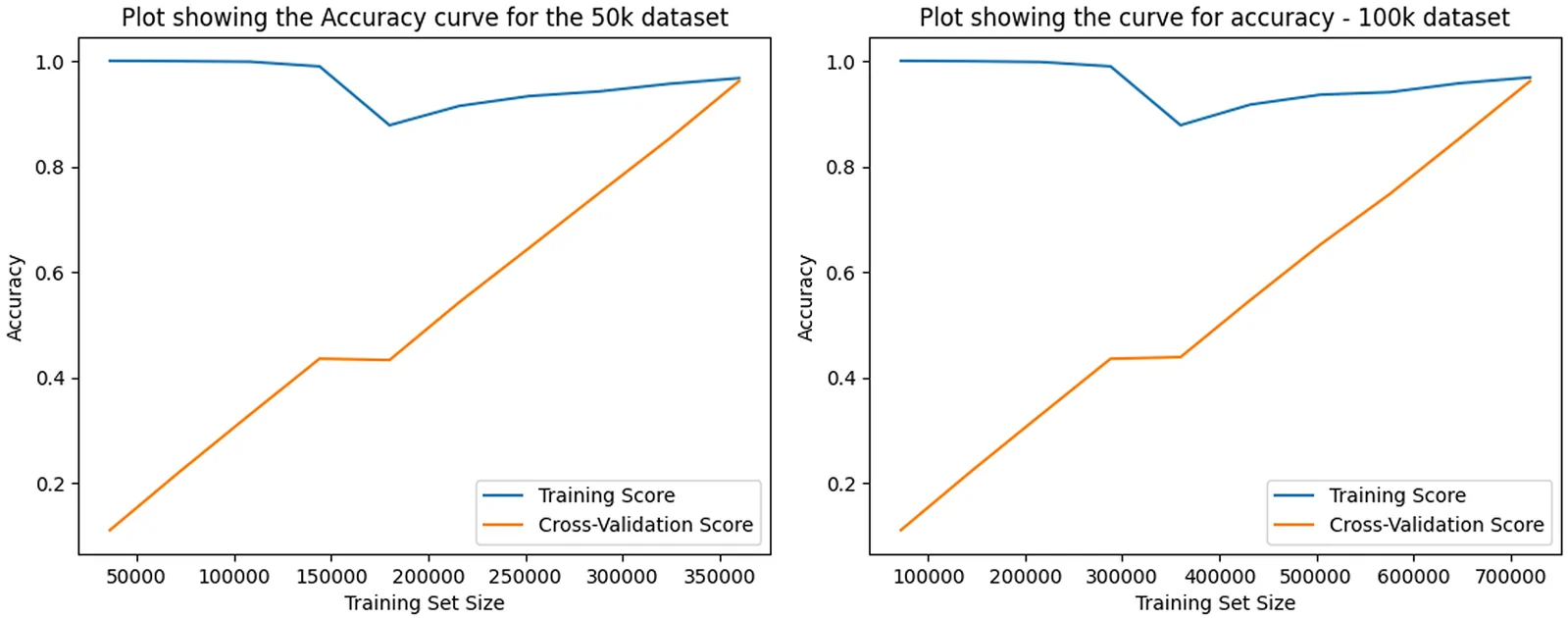
Accuracy curves for training and testing using the basic Random Forest algorithm on the 50k and the 100k datasets.

The learning curves against the running of the model with the two datasets show that the training phase has a linear plateau, indicating the efficiency of the training of the model. During the testing phase, the curve begins at the minimum and gradually reaches the level of the training phase. This indicates that the learning curve was gradual, and the final accuracy was achieved, which is in line with the testing data. If the testing curve had ended at a lower or higher level than the training curve, it would be a case of underfitting or overfitting, respectively ( Figure 3). Even with the increase in the dataset, there was no underfitting or overfitting seen with the model, and the engineered features and the application of PCA have well balanced the importance of the features represented within the original CICDDoS2019 dataset.

This model showed a consistent performance when used against both the 50k and 100k datasets. To test this method of creating an efficient feature engineered dataset, other algorithms, such as Gradient Boost, Logistic Regression, SVM, and basic deep learning models, such as neural networks, were tested. The dataset showed consistent performance in all these models with minimal possible configuration changes, which kept them simple and less burdensome on the computational power required for their execution. All these models were also tested against both the 50k and 100k datasets. The performance was consistent against all these models, and this was observed using the learning curves run for each of these models ( Figure 3). All the models showed a consistent learning curve against both datasets and finally reached a peak near the training curve. This demonstrates the efficiency of the model under varying circumstances, which is expected in large networks with multiple IoT devices communicating with each other. Another major concern is the dynamic nature of these communications, which is addressed by the feature engineering step, and PCA, which efficiently handles the variability in the dataset by reducing the dimensionality of the varying features. With F1-scores over 0.90, recall over 0.91, and precision over 0.95 on average across multiple classes, and across both datasets, this model proved to efficiently handle the dynamic nature of traffic, an essential component of SDNs. This component serves as an important feature when designing intrusion-detection systems for dynamic environments, and this model addresses these requirements.

The scalability and adaptability of this approach are proven by the performance of the model against the increased dataset and multiple algorithms that were tested. This shows the importance of feature engineering and feature categorization along with dimensionality reduction in the effectiveness of the chosen ML or DL model in the classification of DDoS attacks using the chosen dataset.

**Figure 4.**
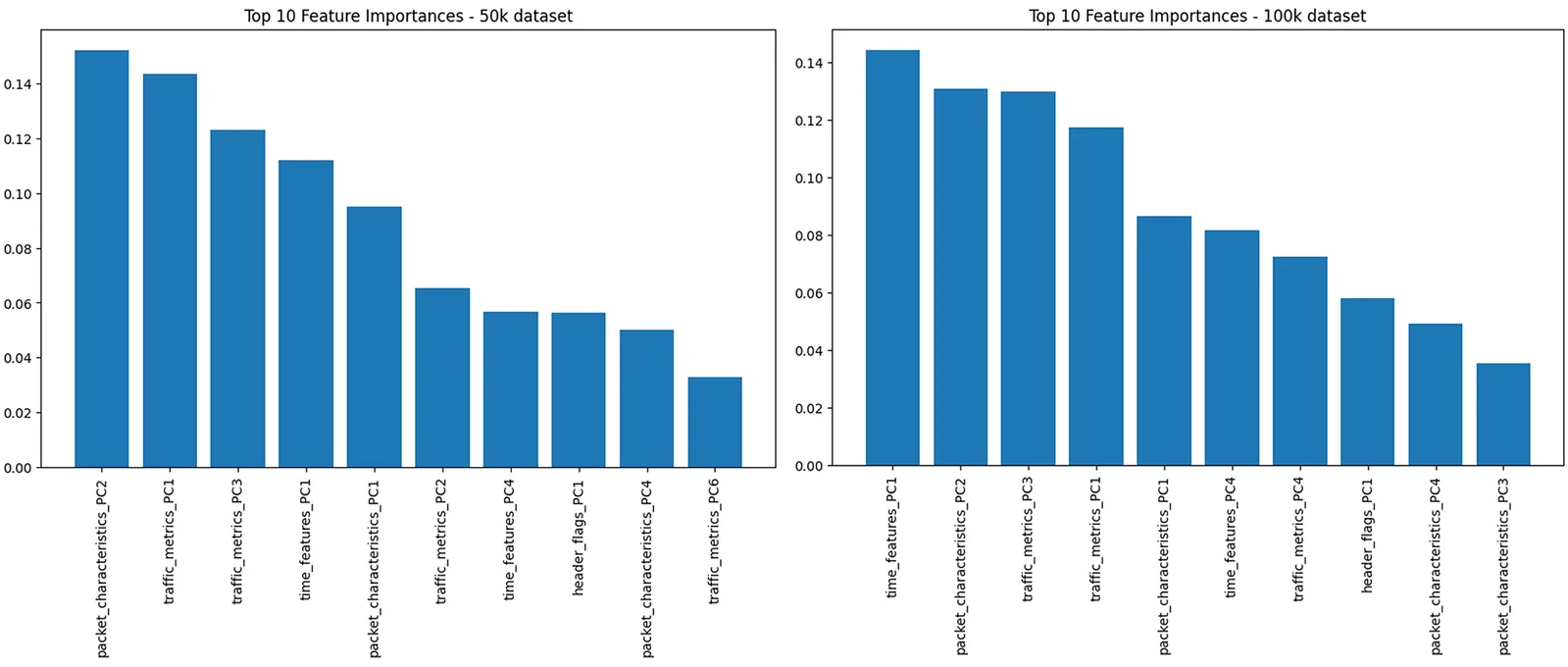
Top 10 features for each of the datasets (50k and 100k).

Of the multiple features involved in the efficiency of the model, a list of the important features responsible for this was analyzed. It was observed that most of the newly generated features were included within the top 10 features based on their importance ( Figure 4), which were observed in both the 50k and 100k datasets. The PCA values that represent the newly generated features, which in turn represent the multiple features categorized under them, have been shown to contribute to the success of the model’s performance.

## 6. Conclusion

By combining feature engineering, dimensionality reduction using PCA, and machine learning for the classification of benign from attack traffic in the CICDDoS2019 dataset, the following conclusions can be drawn from this study. The pivotal concept of feature engineering, capable of capturing the domain-specific nuances of the features within the network traffic, has been a key feature in this efficient classification. In addition to this engineering and processing step, the application of PCA to these derived dataset categories and adding them to form the new dataset led to a significant reduction in the computational burden. This concept has shown its efficiency not only in the Random Forest model that showed robustness, but also against multiple machine learning models such as Gradient Boost, Logistic regression, SVM, and the basic DL model, the neural network in the primary dataset chosen for this study. Thus, this model proves to be a reliable and scalable approach for efficient detection of DDoS attack traffic in SDN traffic data.

## 7. Future work and recommendations

This work could be enhanced by combining advanced models using ensemble techniques, which is an upcoming and promising field that can exploit the advantages of multiple models. Class imbalance has been a major concern in datasets, such as this case, when considering real-world scenarios. This could be improved and addressed to increase the performance of the selected models. Finally, the use of time-series models or a combination of approaches using hybrid approaches could help capture the temporal dependencies present within the network flows, which could in turn improve the accuracy of classification.

## Ethical considerations

Ethical approval and consent were not required.

## Data Availability

The data supporting this study are freely available at https://www.unb.ca/cic/datasets/ddos-2019.html last accessed at 31.12.2024. Basic identification details such as Name, Email, Organization, Job title, and country need to be provided for the provider’s internal statistical purpose, and entire dataset can be downloaded.
