# Applying Principal Component Analysis for Categorized Dimensionality Reduction in DDoS Detection for Software-Defined Networks

**DOI:** 10.12688/f1000research.163778.2

**Published:** 2026-07-27

**Authors:** Keerthana Balaji, Mamatha Balachandra

**Affiliations:** 1 Manipal School of Information Sciences, Manipal Academy of Higher Education, Manipal, Karnataka, India; 2 Manipal Institute of Technology, Manipal Academy of Higher Education, Manipal, Karnataka, India

**Keywords:** DDoS attack, Software Defined Network, CICDDOS2019 dataset, Machine Learning, Feature Engineering

## Abstract

**Background:**

The explosive growth of Software-Defined Networks (SDN) has introduced unmatched scalability with increased flexibility, an essential component of this modern, complicated network infrastructure. While machine learning models promise to be a viable approach for detecting Distributed Denial of Service (DDoS) attacks, their efficiency relies on the quality of the engineered features.

**Methods:**

In this study, an innovative approach for categorizing newly generated features based on domain-specific relevance is applied, followed by Principal Component Analysis (PCA) on each of the categories for dimensionality reduction. These new engineered features represent the originality of the features within the original dataset without losing their integrity by dropping multiple features from the original dataset. These PCA-transformed features, along with other individual features that were not used in the previous step, were merged into a single dataset for further processing using Machine Learning classifiers. This unique methodology not only addresses the curse of dimensionality but also ensures that the meaningful variance within the categories of features is retained. The CICDDoS2019 dataset was used to evaluate the developed model against features engineered from this dataset. Performance was evaluated using accuracy, precision, recall, F1-score, ROC-AUC, log loss, ECE, and cross-validation. The primary dataset comprised of 499,998 total samples consisting of nine attack and one benign classes, split into training, validation, and test sets. Each category group retained ≥95% variance, compressing 45 to 27 PCA components. The proposed grouped PCA pipeline, with all transformers fitted on the training partition, achieved a weighted F1-score of 0.9991, AUROC of 1.0000, and mean ECE of 0.000276 on the primary dataset, improving further to 0.9994, 1.0000, and 0.000202, respectively on a doubled dataset, with five and ten-fold cross-validation confirming strong generalisability and scalability across both scales.

**Conclusion:**

This planned and logically structured approach underscores the importance of domain-driven feature generation and categorization.

## 1. Introduction

The transformation of network management through the introduction of Software Defined Networking has advanced the field of networking, and one of its benefits is the separation of the control balance from the data plane. This enables better handling of traffic, provides opportunities for scalability, and improves overall efficiency. Despite these benefits, the centralized nature of Software Defined Networking renders it vulnerable to Distributed Denial of Service (DDoS) attacks. Situations of such attacks require utmost attention as the attacker floods the controller with malicious traffic, with potential targets being the disruption of network operations and the availability of online services. Conventional security approaches often fall short of the growing sophistication of DDoS threats, which highlights the requirement for advanced techniques such as machine learning (ML), which could be an effective solution for detection and mitigation. ML algorithms analyze network traffic patterns to classify malicious behavior; however, their performance depends on the quality of the input features. High-dimensional network traffic datasets contain redundant, noisy, and irrelevant information, necessitating feature selection and engineering to create meaningful features from raw data, such as packet headers, flow durations, and traffic volumes, ultimately improving the accuracy and generalization of models for DDoS attack detection. ^1, 2^ Efficient management of the control plane is performed by the controller, and that of the data plane is performed by switches. ^3^ When attackers send requests with a high bandwidth to fill in the offered bandwidth of the target server, the server becomes inaccessible to authentic users. ^4^

Traditional defense mechanisms include firewalls and general Intrusion Detection Systems (IDS), which struggle to detect some DDoS attacks that have recently become increasingly sophisticated. However, the efficiency of ML depends on the quality of features in the input data. ^3, 5^ The high dimensionality seen in network traffic datasets has many features that indicate packet headers, flow durations, traffic volumes, and other features that describe the nature and volume of traffic passing through networks. Although these features provide vital information related to the network, redundancy, noise, and irrelevant information hinder the detection process. The process of selecting appropriate features and engineering new meaningful features from available features is vital in such models. ^6– 8^ Raw features include the data obtained from the traffic from the basis for the input features used in ML models. ^9– 11^ Such features play a key role in improving the capacity of a model to detect attacks against systems and networks, and they are also good at generalization. ^6, 7, 12^

Machine learning is capable of quickly classifying attack traffic from benign traffic (binary), but the complicated forms of DDoS attacks owing to advanced evasive techniques with multiple types of traffic combined in a single attack can be challenging. ^13^ To combat such complicated attacks, researchers have used Computational Neural Networks (CNN), a deep learning model, and have considered Recurrent Neural Networks (RNN) that are efficient in traffic classification. ^14– 16^ Effective principles such as Principal Component Analysis (PCA) have proven to be very effective in addressing many issues, particularly the curse of dimensionality, by transforming higher-dimensional data into lower-dimensional data.

The model created in this study was evaluated using the CICDDoS2019 dataset, which is publicly available and used by many DDoS detection studies. Data preprocessing was performed according to the proposed methodology and used to train ML models, such as Gradient Boosting Machines, Random Forest, and Neural Networks. The performance was compared against globally transformed features using PCA, with results demonstrating significant improvements in the accuracy and robustness of the simple ML model.

The work done with the proposed innovative technique for feature engineering and classification using a simple ML model is presented in the following sections. Section 2 focuses on the literature related to DDoS detection and dimensionality reduction along with the techniques used for the same. Section 3 outlines the proposed methodology, which includes categorization of features and PCA-based dimensionality reduction. Section 4 explains the setup and results of the experiments, along with a discussion of the results. Section 5 summarizes the output of the experiment to conclude and extends the research directions for future work.

## 2. Related work

Abdulhammed et al. ^17^ explored the improvement of IDS using ML. The focus was primarily on reducing the dimensionality of the CICDDoS2017 dataset using autoencoders and PCA to enhance the performance of the classifier. Classifiers, such as Random Forest, Linear Discriminant Analysis, and Quadratic Discriminant Analysis, were tested. This was followed by a new performance metric, CombinedMc, to evaluate the classification of multiple classes better. This study achieved an accuracy of 99.6%, along with other key metrics, by significantly reducing the feature dimensions. Distribution-based balancing was used in this study to address the imbalance between classes.

A proactive feature selection model with nature-inspired optimization algorithm was used by Riydh et al. ^18^ for the selection of relevant features in the CICDDoS2019 dataset. Algorithms such as Random Forest (RF), K-Nearest Neighbour (KNN), and Support Vector Machine (SVM) were implemented for the classification of normal traffic from malicious traffic. ^19, 20^ This model is shown to outperform existing methods in terms of the detection rate, overall accuracy, and reduction in false positives. This shows the importance of the feature engineering step for machine learning processing and its contribution to the performance of ML algorithms.

Another study highlighting the importance of feature engineering was conducted by Pegah et al., ^21^ wherein the proposed model worked on an Ensemble Feature Selection method with a multi-aspect perspective. The relevant features are selected based on each type of attack, along with a combination of statistical filtering techniques and machine learning algorithms. Prediction times can be reduced by focusing on key features in the dataset, which increases the performance of the ML algorithm. This, in turn, improves the mitigation capabilities of the algorithm. A more informative and precise representation of the traffic data adds to the overall performance of the algorithms.

In a review by Muhammed et al., ^22^ the importance of ML power for the detection of DDoS attacks was summarized based on references from numerous studies. This summary highlights the significant impact of the appropriately chosen dataset and the features selected from the dataset. This step helps researchers and practitioners develop robust solutions for handling DDoS attacks.

A deep learning-based approach that leverages the advantages of RNN and LSTM ML models was developed by Jiyeon et al. ^23^ The N-BaIoT dataset was used in this study, which simulates botnet attacks on multiple IoT devices. Approximately 115 features presented within the dataset were categorized into five groups, and the primary key was based on the time window, which provided the best performance.

In another study by Muhammad et al. ^24^ conducted as a systematic review, the detection of DDoS attacks was focused on backward elimination, chi-squared tests, and information gain scores for the creation of datasets with significant features. This optimization was shown to increase the efficiency of the tested machine learning models, which were fine-tuned and tested. A feature reduction of up to 68% was achieved, with a minimal accuracy loss of 003%. This strategic combination of feature and machine learning was validated using cross-validation and AUC analyses to mitigate overfitting and collinearity. Among the various algorithms, K-nearest neighbors (KNN) performed the best overall, followed by SVM. Random Forest (RF) performs well on low-dimensional datasets with discrete features, although it is simple and quicker than the others.

Various studies have chosen the best features to improve the performance of the chosen ML model. The important works related to feature engineering used in DDoS attack detection is summarized in Table 1. Most of them have binary classification of attacks and benign traffic. Some studies in the literature have proposed models that perform better with both binary and multi-class classification, but their performance is less than that of binary classification. Based on the literature and data compilation from multiple studies, the following research gaps were identified:

**Table 1.** Important works highlighting feature engineering in DDoS detection.

S.No.	Dataset used	Feature engineering	Feature reduction	Technique used	Class labelling	Accuracy	Reference
1.	IoT-CIDDS	Yes	Yes	5 different ML algorithms	Single	-	^6^
2.	CICDDoS2019	No	Yes	CNN/BiLSTM	Binary	94.52%	^31^
3.	CICDDoS2019	No	Yes	RF, DT, ADA, XGB, MLP, DNN	Binary	99.97%	^11^
4.	CICDDoS2019	No	Yes	GB, ADA, CB	Binary Multi	99.3% 97%	^20^
5.	CICDDoS2019	No	Yes	RF	Binary	99.99%	^19^
6.	CICIDS2019 and CICIDS2017	No	Yes	DT, MLP, XGB, RF	Binary Multi	99.2% 98.83%	^16^
7.	CICIDS 2017 and CICDDoS 2019	No	Yes	RF *, GB, Weighted Voting Ensemble (WVE), KNN, LR	Binary	99.0%	^4^
8.	CICDDoS2019	No	Yes	RF *, LGB, CatBoost, CNN	Binary	99.9%	^12^
9.	CICDDoS2019	No	Yes	RF, ANN, KNN, BNN	Binary	99.7%	^32^
10.	CICDDoS2019 KDD-CUP1999	No	Yes	CNN *, SGD, DT, RF	Binary	98.0%	^3^
11.	CICDDoS2019	Yes	Yes	RF	Multi	97.0%	-

* Best performing algorithm in each study.

## 3. Research gaps

1. The implemented feature selection techniques drop certain features that could possibly misrepresent the importance of the class they represent.
2. Binary classification using such selected features has a limited ability to classify modern multi-class types of complicated attacks.
3. The importance of engineering new features from existing ones could improve the representation of data for the efficient classification of various types of traffic.

## 4. Setup used for the experiment

**Figure 1.**
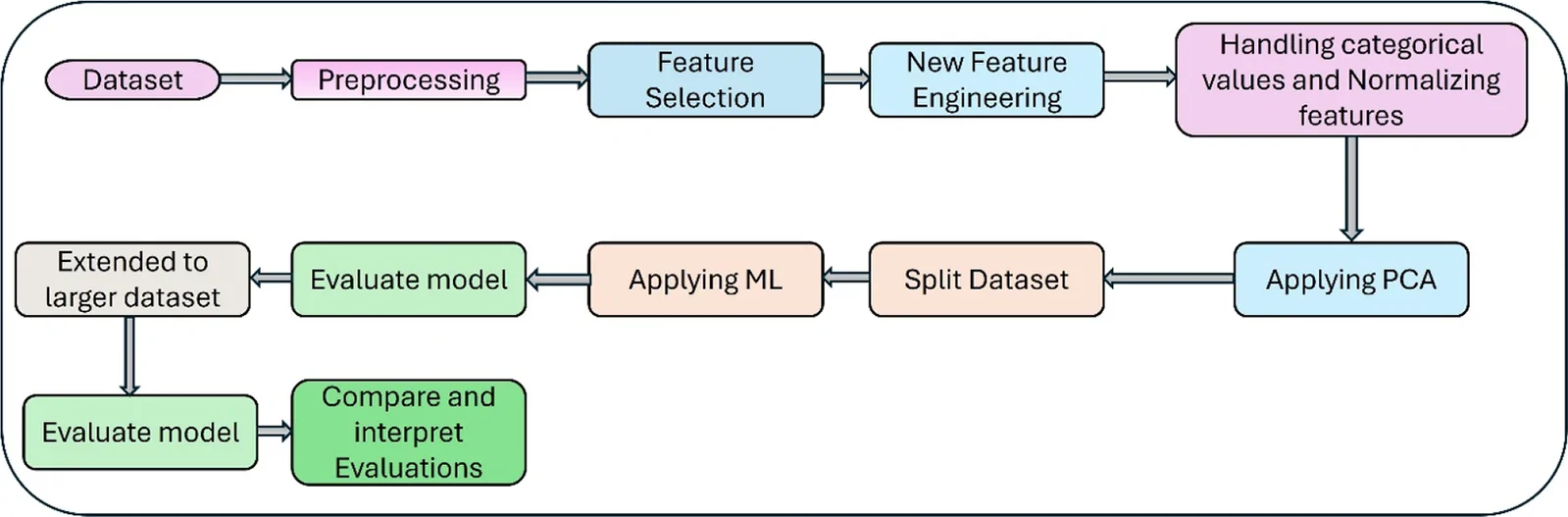
Shows the flow of work done in this study.

Here, the details of the dataset used, the new features engineered, the features dropped, and the detection algorithm used are described. In Figure 1, the steps used in this study, including feature selection, feature engineering, splitting the dataset, and the rest of the steps, are summarized.

### 4.1 Dataset description

The CICDDoS2019 dataset used in this study was published by Sharafaldin et al., ^25^ which is a good fit for testing models developed to detect DDoS attacks in SDNs *.* In this dataset, created using actual traffic, there are more than 80 features, which form a good benchmark dataset for use in DDoS attack detection studies. There are multiple types of attacks that utilize TCP/UDP protocols. Compared to older datasets, this dataset sets a benchmark that includes 12 different DDoS attack types, with categories as reflection- or exploitation-based.

### 4.2 Data preprocessing

Before training the model, preprocessing of the dataset is an essential step for removing noise and reducing redundant and unnecessary data. This step is crucial and helps increase the efficiency of the model performance by reducing the complexity caused by the features within the dataset.

4.2.1 Handling missing and null values

Missing and null values affect the accuracy and precision of the model’s efficiency. In this study, the missing values were replaced as null values and all the null values were then imputed with ‘0,’ to maintain uniformity which improves the performance of the model.

4.2.2 Feature selection and engineering

In our study, this was the most important step, as the design aimed to identify the best features contributing to the classification task. New features are generated by combining as many features as possible and passing them to the model without compromising its efficiency. The following equations show the combinations of features for generating new features:

In this step, a total of 42 features were combined logically and reduced to 15 new features. This reduction of feature sets (72 original features reduced to 45 features (15 new features generated + 30 original features) in total reduced the complexity of computation of the model considerably and contributed to the performance of the model. After generation of these new features, the 42 original features used in the creation of these 15 new features were dropped before further processing of the dataset.

**Importance of the new features generated:**

1. **Avg Packet Size:** The average packet size was calculated by dividing the total length of packets by the total number of packets.
2. **Total Packets:** This represents the total number of packets in a flow by summing the total forward and backward packets.
3. **Total Bytes:** This gives the total number of bytes transferred in a flow by summing the total length of forward and backward packets.
4. **Average Packet Length:** The average packet length was calculated by averaging the mean packet length, mean length of the forward packet, and backward packets.
5. **Flow IAT Aggregate:** The various inter-arrival time statistics flow IAT mean, std, max, and min were used to provide a comprehensive overview of the timing patterns in the flow.
6. **Fwd IAT Aggregate:** By combining various forward inter-arrival time statistics, the forward IAT mean, std, max, and min provide a comprehensive overview of the timing patterns in the forward traffic.
7. **Bwd IAT Aggregate:** Here various backward inter-arrival time statistics, backward IAT mean, std, max, and min, are combined to provide a comprehensive overview of the timing patterns in the backward traffic.
8. **Total Header Length:** The total header length of packets in a flow was calculated by summing the forward and backward header lengths.
9. **Total Segment Size:** This feature calculates the total segment size of packets in a flow by summing the average forward and backward segment sizes.
10. **Subflow of Total Packets:** This feature calculates the total number of packets in the subflows by summing the forward and backward subflows.
11. **Subflow Total Bytes:** This feature represents the total number of bytes in the subflows by summing the forward and backward subflow bytes.
12. **Flag Aggregate:** This feature combines various flag counts and SYN, RST, ACK, URG, and CWE Flags to provide information about the type of traffic and potential attacks.
13. **Flow Speed Ratio:** This feature calculates the ratio of bytes per second to packets per second, providing insights into the efficiency of the flow. It uses flow bytes and packet features.
14. **Active Time Aggregate:** This feature combines various active time statistics, active mean, std, active, and min, to provide insights into the periods of active data transfer.
15. **Idle Time Aggregate:** This feature combines various idle time statistics Idle mean, std, max, and min to provide insights into the periods of inactivity in the flow.

4.2.3 Handling categorical values

The Label column describes the type of attack and its multiple labels, which are designated as target columns. These values were converted to numerical values using the LabelEncoder from the Scikit library. Each of these values was assigned a numerical value designating an individual category, and then processed further through the model.

4.2.4 Normalizing features for improved model performance

Normalizing Features is vital in ML, as it ensures that the values of the various features are scaled for equal contributions to the process of learning the model. Implemented StandardScaler and PCA on the training set, and applied to validation and test sets to transform (a constraint enforced automatically through sklearn pipeline objects), thus preserving the integrity of evaluation set.

### 4.3 Importance of applying PCA to the dataset

Processing these categorical features using Principal Component Analysis (PCA) and passing these values into machine learning algorithm for classification into multi-class classifications reduced 45 features in a semantic manner to 27 components. This study consists of two major parts: feature categorization and basic model evaluation for classification.

### 4.4 Splitting of data

Another important step is splitting the data into training and testing sets. The primary dataset (n = 499,998) is first divided in the ratio of 70/30 into a combined train-validation set and a held-out test set. The train-validation set is further split in the ratio of 85/15, and stratified sampling is applied at every split to preserve class proportions. This sampling resulted into final three partitions; training (59.5%, n = 297,498), validation (10.5%, n = 52,500), and test (30%, n = 150,000) sets. The validation set is used exclusively for post-hoc calibration fitting and this was not used earlier to avoid influence on transformer fitting or classifier training. The train_test_split function from scikit-learn 1.7.0 is used with random_state = 42. The Scalers and PCA components were fitted on the training partition and applied via transform to validation and test partitions. The same application of pattern was followed for the doubled dataset (n = 999,999). Tables 3 and 4 show exact per-class counts across all three partitions for both the datasets, respectively.

### 4.5 Application of the algorithm

The RF model was used in this study for this classification task using the libraries for this model. Configured the model with n_estimators = 200 and no maximum depth, which allows trees to grow to the complexity required by the data. Parallelism is enabled via n_jobs = 6 (manually chosen to prevent thermal throttling during continuous multi-hour runs), utilising six of the sixteen CPU threads available on the machine used for this study. The model is trained on the training partition, and the test partition used at evaluation. All experiments were conducted on an AMD Ryzen 7 4800H (8 cores/16 threads, boost 4.2 GHz), 32GB DDR4 RAM, NVIDIA RTX3050 4GB VRAM, running Python 3.10 and scikit-learn 1.7.0. All computations were limited to CPU-only. On the 50k (primary) dataset, training time for the Random Forest on the grouped PCA features was 42.5 seconds with inference completing in 789 ms on the full test set (150,000 samples). Whereas, on the 100k (doubled) dataset the corresponding times were 101.5 seconds and 1,575 ms respectively. The timing results for all pipeline configurations across both dataset scales are reported in Table 6.

### 4.6 Assessment of model performance

The performance of the model was evaluated using the following evaluation metrics: F1 score, Accuracy, Precision, Recall, ROC-AUC score, confusion matrix, and log loss function. A higher ROC-AUC score indicates a better ability to discriminate between multiple classes. The log loss is another important value that indicates the uncertainty of the model in its predictions, where lower values indicate a higher confidence in the predictions. Finally, the confusion matrix provides details of the predictions made by the model and shows the values for each class, allowing us to analyze the specific error types (false positives and negatives). Overall, these metrics provide a better understanding of its advantages and disadvantages, locating potential areas for improvement in making informed decisions. The grouped PCA pipeline produces an identical component structure at both 50k and 100k dataset scales (27 components total), demonstrating structural invariance to training set size is represented in Figure 2. The multi-class AUROC is computed using a One-versus-Rest (OvR) formulation, providing both macro-averaged and weighted-averaged values ( Table 11). The Brier score and Expected Calibration Error (ECE) are computed per class under the same OvR used above, and then averaged. The ECE used ten equal-width probability bins weighted by bin size. The calibration diagnostics are reported as per-class calibration curves ( Figure 2a and Figure 2b) along with an aggregate reliability diagram ( Figure 3). The post-hoc Platt scaling and isotonic regression are evaluated on the validation set ( Figure 4). The stability of the results is checked using five-fold and ten-fold stratified cross-validation, and pairwise model differences are checked using paired Wilcoxon signed-rank tests ( Table 8).

**Figure 2.**
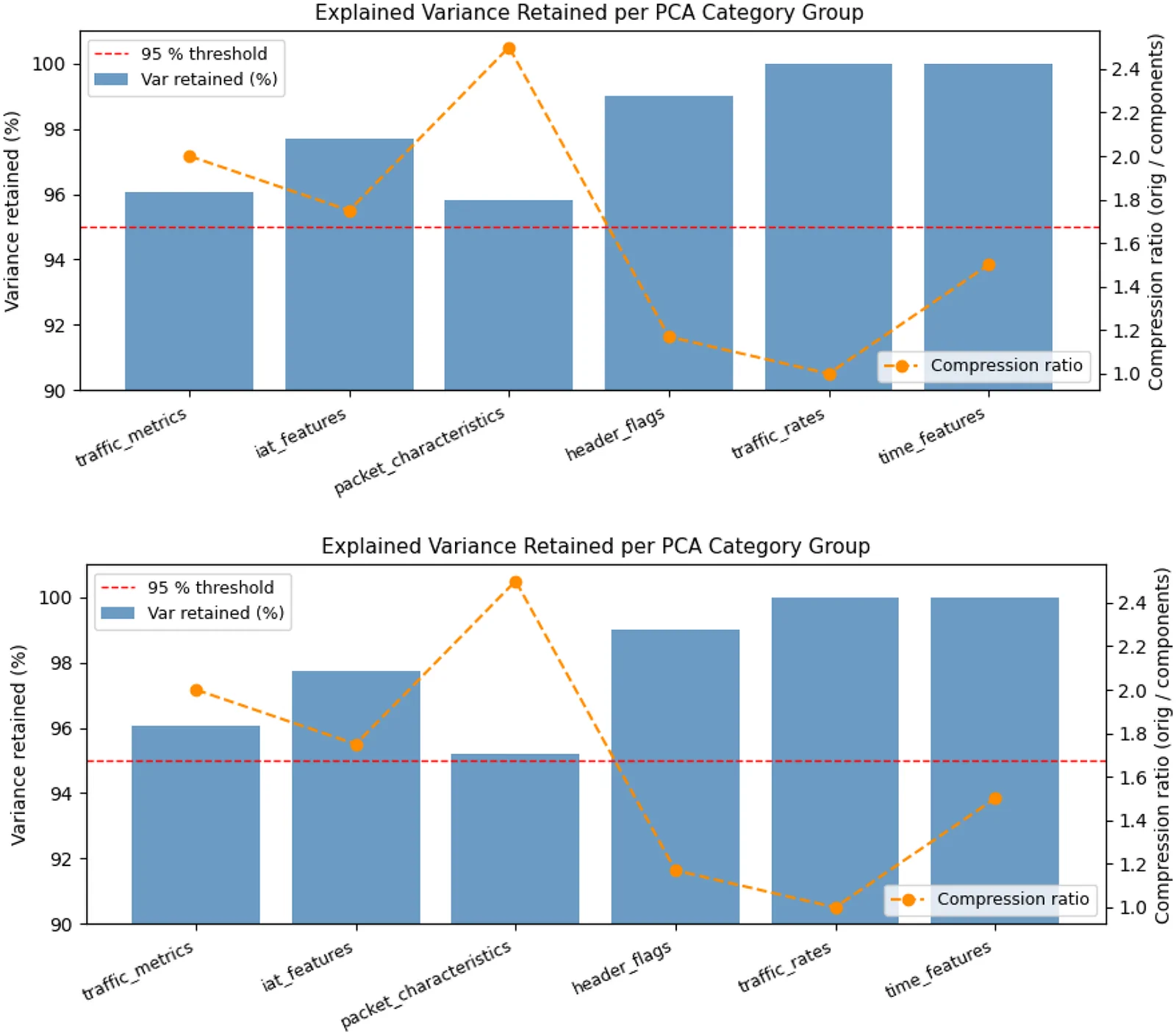
Explained variance retained (%) per PCA category group (bars, left axis) and compression ratio (line, right axis). The dashed red line marks the 95% variance threshold. All six category groups meet or exceed this threshold. The grouped PCA pipeline produces an identical component structure at both 50k and 100k dataset scales (27 components total), demonstrating structural invariance to training set size.

**Figure 2a.**
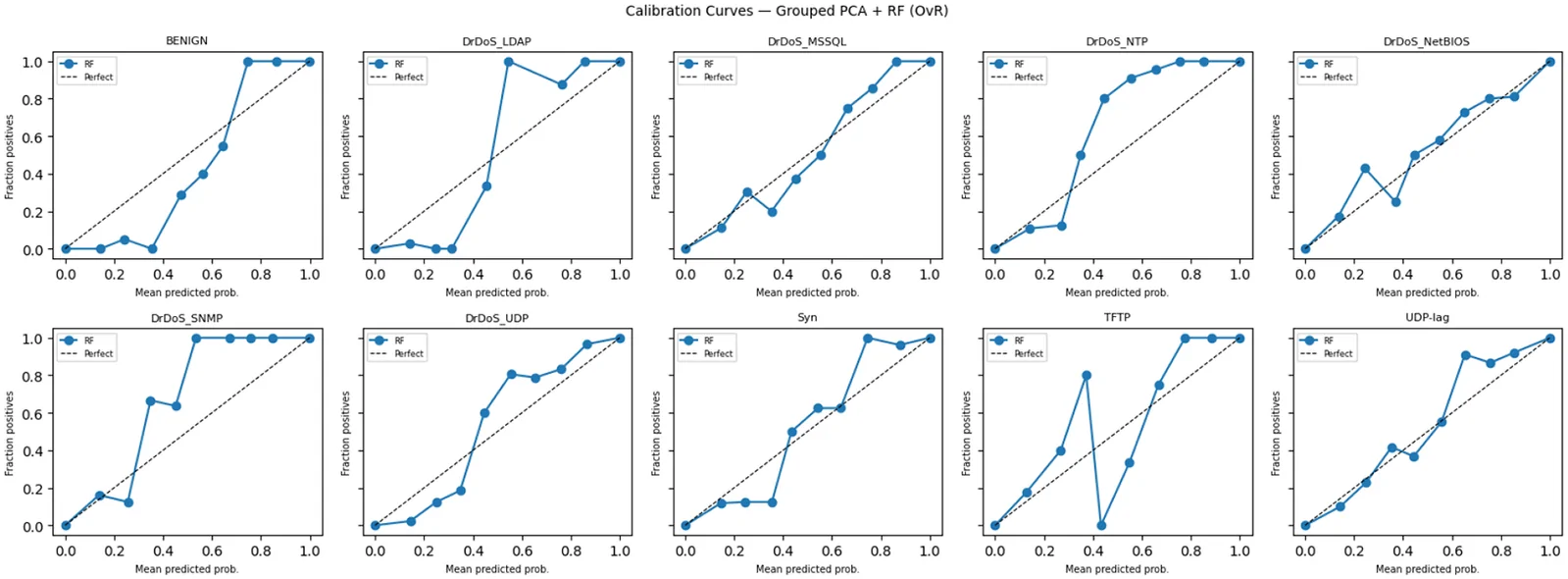
Per-class calibration curves — grouped PCA + Random Forest, 50k dataset (N = 499,998). Calibration curves for all ten attack classes using a One-versus-Rest (OvR) formulation, evaluated on the held-out test set (n = 150,000, 15,000 per class). The solid blue line shows the fraction of true positives per predicted probability bin against mean predicted probability; the dashed line represents perfect calibration. Ten equal-width bins were used. The uncalibrated Random Forest (n _estimators_ = 200) achieves a mean ECE of 0.000276 across all classes, substantially lower than typical values reported in network intrusion detection literature (0.02–0.10). Apparent jaggedness in low-probability bins reflects sparse bin occupancy rather than systematic miscalibration.

**Figure 2b.**
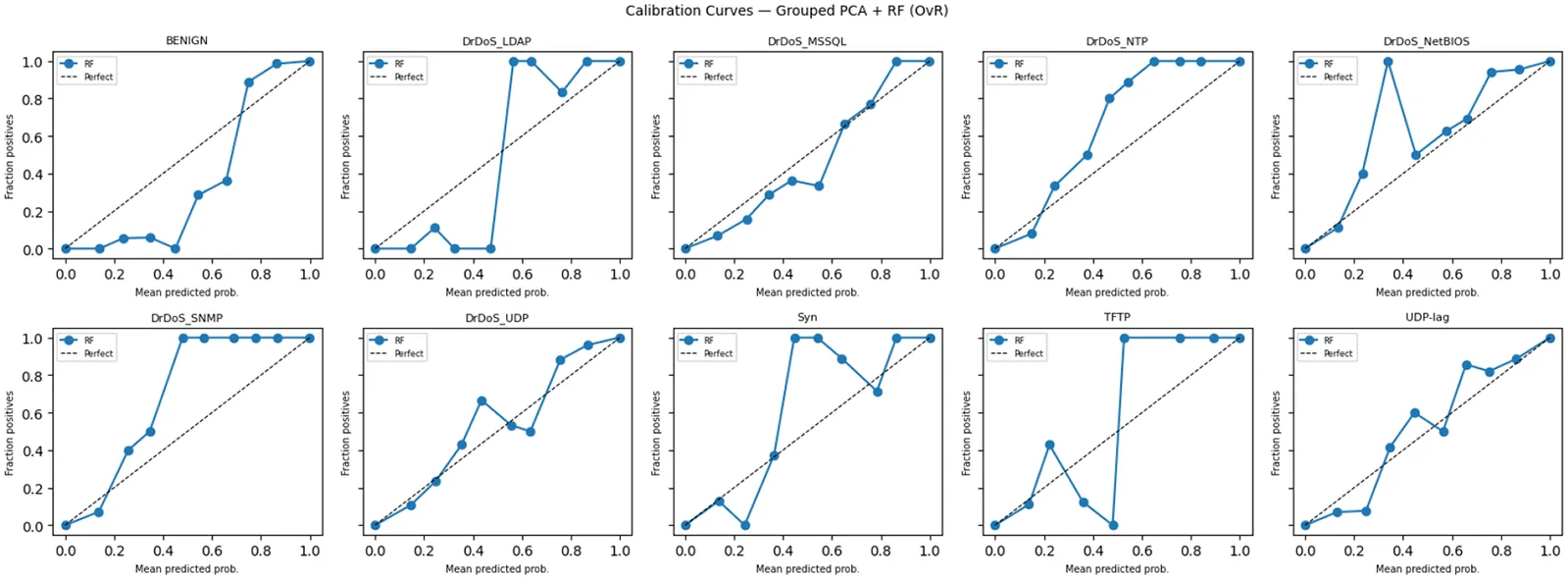
Per-class calibration curves — grouped PCA + Random Forest, 100k dataset (N = 999,999). Calibration curves for all ten attack classes using a One-versus-Rest (OvR) formulation, evaluated on the held-out test set (n = 300,000, 30,000 per class). Settings identical to Figure 2a. Mean ECE improved to 0.000202 at the larger scale without post-hoc correction, confirming that calibration quality scales consistently with dataset size. Curve shapes are visually consistent with Figure 2a, confirming that calibration behaviour is stable across dataset scales — a property attributable to the structural invariance of the grouped PCA decomposition.

**Figure 3.**
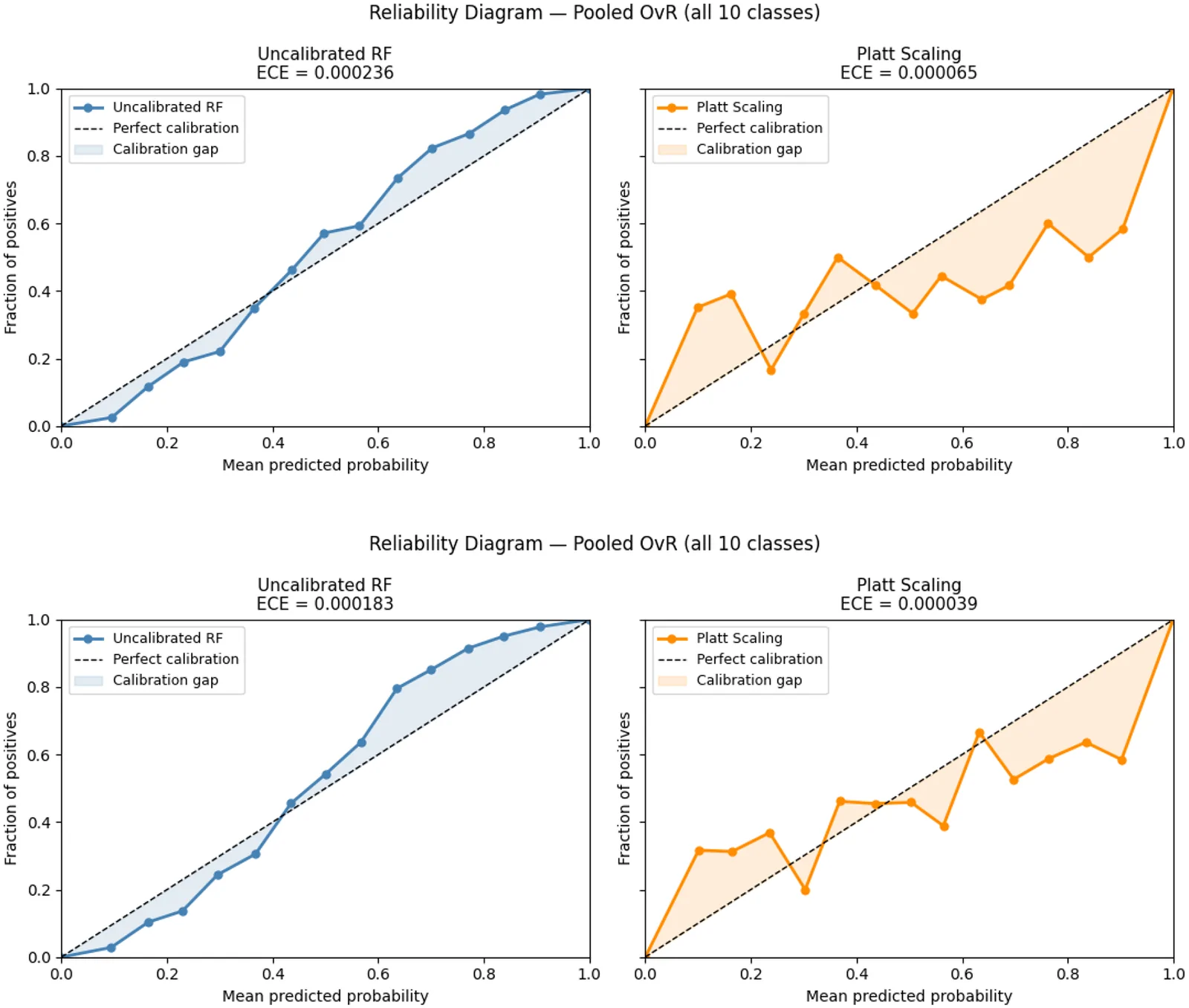
Aggregate reliability diagrams (pooled OvR across all 10 classes). Left: Uncalibrated RF (ECE = 0.000236 at 50k, 0.000183 at 100k). Right: Platt scaling (ECE = 0.000065 at 50k, 0.000039 at 100k). The uncalibrated model tracks the perfect diagonal closely, indicating exceptional native calibration. Platt scaling achieves a lower aggregate ECE but the reliability diagram shows the uncalibrated model provides more stable probability estimates across the prediction range — consistent with known behaviour when isotonic or sigmoid calibration is applied to already well-calibrated models.

**Figure 4.**
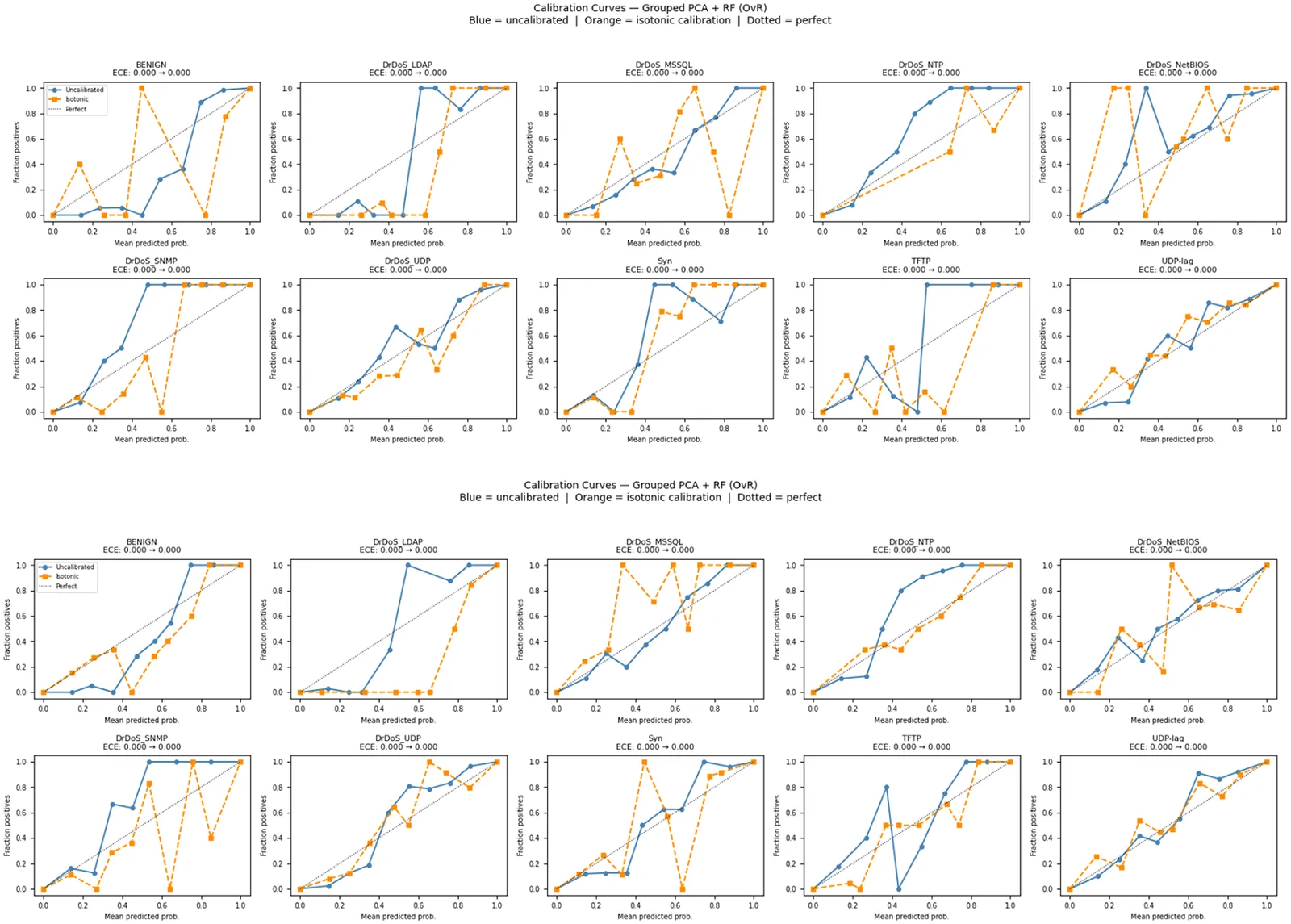
Per-class calibration curves (OvR) for the grouped PCA + Random Forest model. Blue = uncalibrated, Orange = isotonic calibration, Dotted = perfect diagonal. ECE values (before → after isotonic calibration) are shown in each subplot title. All ECE values round to 0.000 at three decimal places, reflecting near-perfect calibration across all ten attack classes at both dataset scales. The consistent curve shapes across 50k and 100k datasets confirm that calibration behaviour is stable with respect to training set size.

## 5. Results and discussion

In this study, the CCIDDoS2019 dataset was used after processing it for feature engineering, where the important features were retained and new features were generated. The final dataset comprises 499,998 samples across 10 attack classes, where eight of the classes contributed 50,000 samples each and rest two classes (DrDoS_MSSQL and DrDoS_UDP) contributed 49,999 each. The terms “50k” and “100k” used in different sections of this paper refer to the per-class sampling size applied during dataset construction, not the total count. The dataset is partitioned into training (n = 297,498), validation (n = 52,500), and test (n = 150,000) sets using stratified sampling to maintain appropriate distribution of classes. The per-class counts for all three partitions are provided in Table 2 (primary dataset) and Table 3 (doubled dataset). The pipeline was applied, with all transformers fitted on the training partition, and the results are presented from the test set ( Table 4). The values from PCA on the features generated, and using these PCA values as part of the dataset considerably reduced the dimensions but retained the interaction and contributions of the features within the dataset. This step reduces the running time of the model with reduced dimensions resulting in less processing time for the model, while retaining the importances of the features. The new features generated considerably improved the classification performance of the model and showed consistency of the model through the evaluation metrics ( Table 5).

**Table 2.** Sample counts across training (59.5%), validation (10.5%), and test (30%) partitions with total N = 499,998. Stratified sampling is applied at every split (random_state = 42).

**Class**	**Train (n)**	**Validation (n)**	**Test (n)**	** Total (n)**
BENIGN	29750	5250	15000	50000
DrDoS_LDAP	29750	5250	15000	50000
DrDoS_MSSQL	29749	5250	15000	49999
DrDoS_NTP	29750	5250	15000	50000
DrDoS_NetBIOS	29750	5250	15000	50000
DrDoS_SNMP	29750	5250	15000	50000
DrDoS_UDP	29749	5250	15000	49999
Syn	29750	5250	15000	50000
TFTP	29750	5250	15000	50000
UDP-lag	29750	5250	15000	50000
**TOTAL**	**297498**	**52500**	**150000**	**499998**

**Table 3.** Sample counts for the doubled dataset with total N = 999,999. Stratified sampling is applied at every split (random_state = 42).

**Class**	**Train (n)**	**Validation (n)**	**Test (n)**	** Total (n)**
BENIGN	59500	10500	30000	100000
DrDoS_LDAP	59500	10500	30000	100000
DrDoS_MSSQL	59500	10500	30000	100000
DrDoS_NTP	59499	10500	30000	99999
DrDoS_NetBIOS	59500	10500	30000	100000
DrDoS_SNMP	59500	10500	30000	100000
DrDoS_UDP	59500	10500	30000	100000
Syn	59500	10500	30000	100000
TFTP	59500	10500	30000	100000
UDP-lag	59500	10500	30000	100000
**TOTAL**	**594999**	**105000**	**300000**	**999999**

**Table 4.** Ablation results across four pipeline configurations. All use Random Forest (n_estimators = 200, no depth cap, n_jobs = 6) with identical train/test splits. Grouped PCA (D) is the only configuration to simultaneously reduce feature count, cut inference time, and maintain accuracy at the level of uncompressed feature sets.

** Configuration**	** Accuracy**	** Macro-F1**	** W-F1**	** AUROC-W**	** Log-Loss**	** # Feats**	** Fit (s)**	** Infer (ms)**
**50k Dataset**								
A — Engineered, no PCA	0.9991	0.9991	0.9991	1.0000	0.0077	45	28.2	1,063
B — Global PCA (0.95 var)	0.9929	0.9929	0.9929	0.9999	0.0342	22	97.9	1,125
**C — Grouped PCA (proposed)**	**0.9991**	**0.9991**	**0.9991**	**1.0000**	**0.0047**	**27**	**49.3**	**931**
**100k Dataset**								
A — Engineered, no PCA	0.9995	0.9995	0.9995	1.0000	0.0052	45	56.9	2,099
B — Global PCA (0.95 var)	0.9948	0.9948	0.9948	0.9999	0.0263	21	216.6	2,019
**C — Grouped PCA (proposed)**	**0.9994**	**0.9994**	**0.9994**	**1.0000**	**0.0026**	**27**	**103.5**	**1,566**

W-F1 = Weighted F1-score. AUROC-W = Weighted OvR AUROC. Timing: AMD Ryzen 7 4800H, n_jobs=6, CPU-only, single run.

**Table 5.** Fit and inference timing across all pipeline configurations at both dataset scales. Inference advantage column shows grouped PCA speed relative to raw features baseline. All experiments: AMD Ryzen 7 4800H (8 cores/16 threads, boost 4.2 GHz), 32 GB DDR4, NVIDIA RTX 3050 4 GB VRAM, Python 3.10, scikit-learn 1.7.0, n_jobs = 6, CPU-only, single run.

**Configuration**	**# Features**	**50k Fit (s)**	**50k Infer (ms)**	**100k Fit (s)**	**100k Infer (ms)**	**Infer advantage vs Config A** *
A — Engineered, no PCA	45	28.2	986	54.2	2,040	baseline
B — Global PCA	22/21	97.9	970	202.4	1,999	+2% faster / −2%
**C — Grouped PCA (proposed)**	**27**	**49.3**	**789**	**103.5**	**1,575**	**+20% / +22% faster**

* Inference advantage: grouped PCA vs raw features baseline. Global PCA component count: 22 at 50k, 21 at 100k (sampling sensitivity). Grouped PCA: 27 at both scales (structurally invariant). GB 10-fold CV runtime: ~4,267s (50k) and ~18,368s (100k) — single-threaded only, n_jobs not supported.

Observations from this study include feature engineering as a key component and critical component in the enrichment of the dataset. The features derived, such as total packets, Flow IAT aggregate, Subflow Total Packets, and Flag aggregate, encapsulate domain-specific knowledge. This concept increases the representational power of the dataset and the ranking of these features from both the datasets used is presented as a Table ( Table 6). The importance of feature engineering, which can retain the representation of the original dataset, has been given considerable importance in previous studies on machine learning for DDoS attacks. ^26– 28^ Additional aggregated metrics, such as Active Time Aggregate, Idle Time Aggregate, enabled the model to effectively capture temporal and flow-based behavioral patterns within network traffic.

**Table 6.** Features – Importance by ranking in the model.

50k dataset Feature: Importances	100k dataset Feature: Importances
• packet_characteristics_PC2: 0.1523 • traffic_metrics_PC1: 0.1436 • traffic_metrics_PC3: 0.1231 • time_features_PC1: 0.1121 • packet_characteristics_PC1: 0.0952 • traffic_metrics_PC2: 0.0653 • time_features_PC4: 0.0567 • header_flags_PC1: 0.0564 • packet_characteristics_PC4: 0.0499 • traffic_metrics_PC6: 0.0327	• time_features_PC1: 0.1444 • packet_characteristics_PC2: 0.1310 • traffic_metrics_PC3: 0.1299 • traffic_metrics_PC1: 0.1174 • packet_characteristics_PC1: 0.0865 • time_features_PC4: 0.0816 • traffic_metrics_PC4: 0.0724 • header_flags_PC1: 0.0581 • packet_characteristics_PC4: 0.0490 • packet_characteristics_PC3: 0.0355

Running PCA to each of the categories separately ensured the high-dimensional space within the features was transformed into a compact representation while retaining the most meaningful variance in each category group. Each category retained ≥95% of its variance: traffic_metrics retained 96.06% (12 features = 6 components), iat_features 97.71% (7 features = 4 components), packet_characteristics 95.82% (10 features = 4 components), header_flags 98.99% (7 features = 6 components), traffic_rates 100% (3 features = 3 components), and time_features 100% (6 features = 4 components) among all the features used in this categorization ( Table 7). Overall, 45 features were compressed to 27 PCA components resulting in a 1.67× (overall) reduction, while retaining the dominant variance structure of each semantic group. The variance breakdown of each component and compression ratios for each category are reported in Table 7. This efficient categorization of features and combining them in their individual grouped PCA components resulted in a feature set proving to be highly discriminative that are evident in the results from evaluation ( Table 4). Retaining the importance of features and reducing noise are essential components of ML algorithms. These algorithms are sensitive to noise and reducing them to the maximum possible extent is an important step in these models. ^29– 32^

**Table 7.** Variance retained and compression ratio per category group. Component counts are identical at both dataset scales, confirming structural invariance. Maximum variance difference between scales: 0.60% (packet_characteristics).

**Category**	**Original features**	**PCA components**	**Var retained 50k (%)**	**Var retained 100k (%)**	**Compression ratio**
traffic_metrics	12	6	0.96	0.96	2.00x
iat_features	7	4	0.98	0.98	1.75x
packet_characteristics	10	4	0.96	0.95	2.50x
header_flags	7	6	0.99	0.99	1.17x
traffic_rates	3	3	1	1	1.00x
time_features	6	4	1	1	1.50x
**TOTAL / OVERALL**	**45**	**27**	**≥95% all groups**	**≥95% all groups**	**1.67x**

Note: Global PCA (not shown) produced 22 components at 50k and 21 at 100k — changing dimensionality between scales. Grouped PCA produced 27 components at both scales.

**Figure 5.**
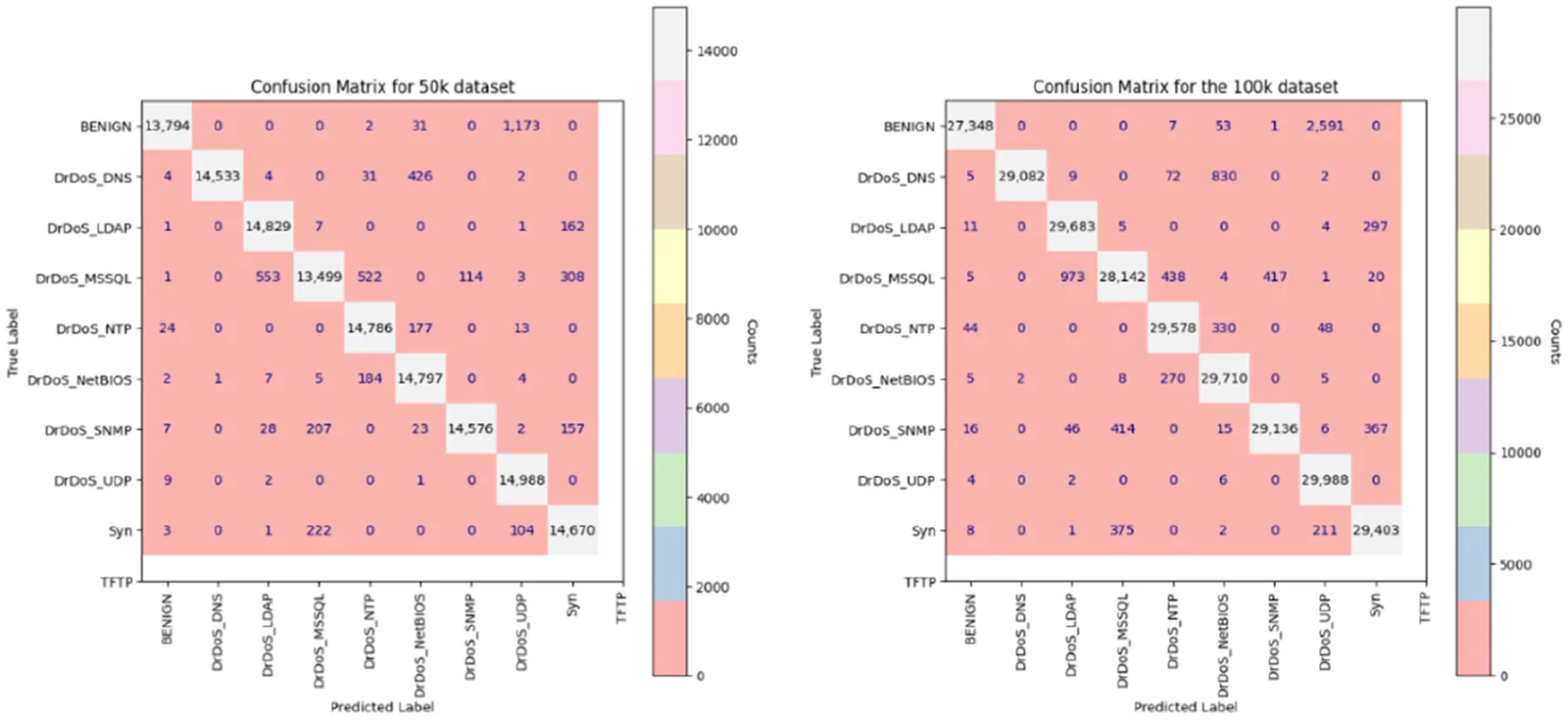
Confusion matrix from the output of running the two different datasets (50k and 100k) using the Random Forest classifier.

In machine learning models, performance against multi-class target sets is an essential feature, depending on the field of interest. ^33, 34^ In areas such as DDoS detection, the capability of the model plays a major role in real-world scenarios. Our model achieved the highest possible accuracy in classifying the various classes within the target column, and this could primarily be a result of the retention of importance within the dataset, although the dimensions were reduced categorically using PCA. This capability is evident from the distribution observed within the confusion matrix, where the false positives are minimal compared to the large size of the datasets used in this study. The confusion matrix for both the datasets (50k and 100k) is shown in Figure 5. This novel concept of categorized PCA successfully retained the importance of the dataset values. The stratified train-test split handled the bias in the evaluation and ensured the class proportions within the training and testing datasets, mirroring the distribution in the original dataset.

**Figure 6.**
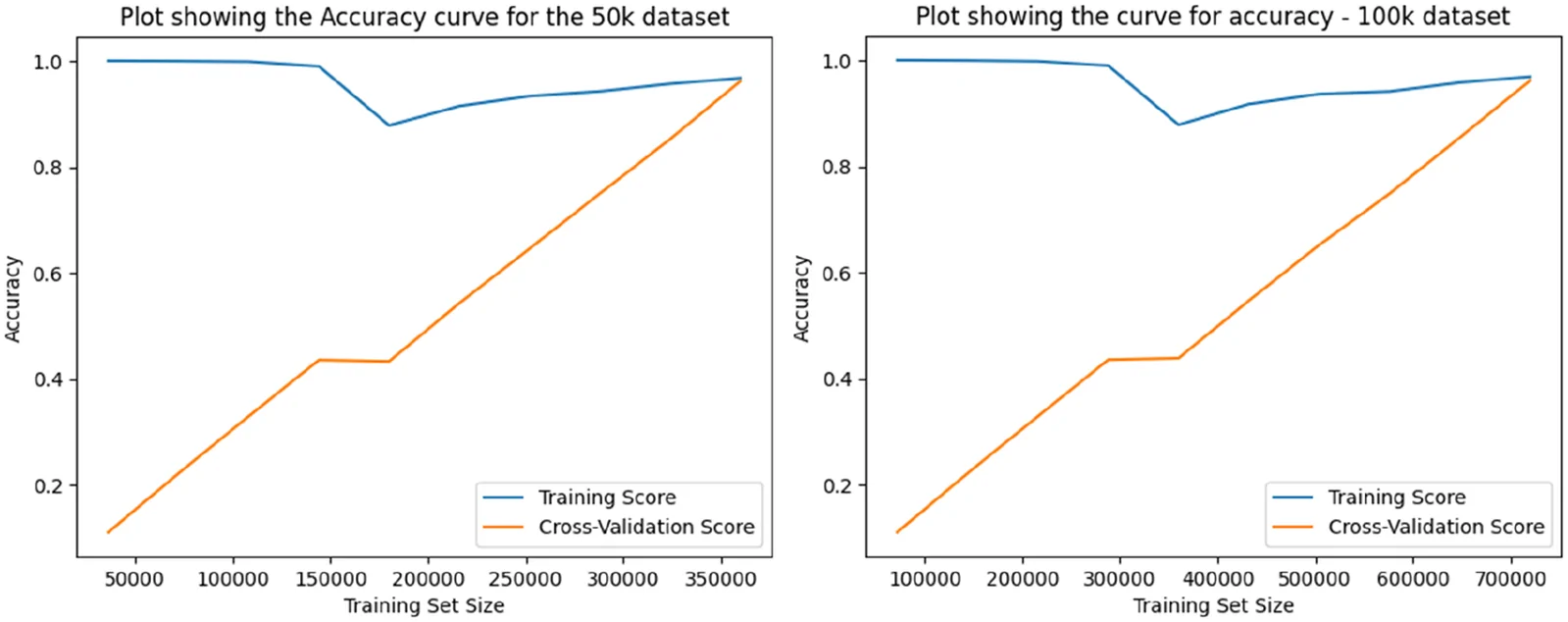
Accuracy curves for training and testing using the basic Random Forest algorithm on the 50k and the 100k datasets.

The learning curves against the running of the model with the two datasets show that the training phase has a linear plateau, indicating the efficiency of the training of the model. During the testing phase, the curve begins at the minimum and gradually reaches the level of the training phase. This indicates that the learning curve was gradual, and the final accuracy was achieved, which is in line with the testing data. If the testing curve had ended at a lower or higher level than the training curve, it would be a case of underfitting or overfitting, respectively ( Figure 6). Even with the increase in the dataset, there was no underfitting or overfitting seen with the model, and the engineered features and the application of PCA have well balanced the importance of the features represented within the original CICDDoS2019 dataset.

This model showed a consistent performance when used against both the 50k and 100k datasets. To test this method of creating an efficient feature engineered dataset, other algorithms, such as Gradient Boost, Logistic Regression, SVM, and basic deep learning models, such as neural networks, were tested ( Table 8). The dataset showed consistent performance in all these models with minimal possible configuration changes, which kept them simple and less burdensome on the computational power required for their execution. All these models were also tested against both the 50k and 100k datasets. The performance was consistent against all these models, and this was observed using the learning curves run for each of these models ( Figure 6). All the models showed a consistent learning curve against both datasets and finally reached a peak near the training curve. This demonstrates the efficiency of the model under varying circumstances, which is expected in large networks with multiple IoT devices communicating with each other. Another major concern is the dynamic nature of these communications, which is addressed by the feature engineering step, and PCA, which efficiently handles the variability in the dataset by reducing the dimensionality of the varying features. With precision, recall, and F1-score of 1.00 across all ten attack classes at both dataset scales, the model demonstrates robust and consistent classification performance, confirming its ability to handle the dynamic nature of multi-class traffic in SDN environments. This component serves as an important feature when designing intrusion-detection systems for dynamic environments, and this model addresses these requirements.

The scalability and adaptability of this approach are proven by the performance of the model against the increased dataset and multiple algorithms that were tested ( Tables 8 and 9). This shows the importance of feature engineering and feature categorization along with dimensionality reduction in the effectiveness of the chosen ML or DL model in the classification of DDoS attacks using the chosen dataset.

**Table 8.** Model comparison across four classifiers on grouped PCA features (27 components). CV-F1 from 5-fold stratified cross-validation through the full leakage-safe pipeline. Fit and inference timing on AMD Ryzen 7 4800H, n_jobs = 6, CPU-only.

**Model**	**Hyperparameters**	**Acc.**	**Mac-F1**	**W-F1**	**CV-F1**	**CV-Std**	**AUROC-W**	**Log-Loss**	**Fit (s)**	**Inf (ms)**
**50k Dataset — 5-fold CV**										
**Logistic Regression**	C=1, lbfgs, multinomial	0.9658	0.9659	0.9659	0.9654	0.0009	0.9978	0.1542	45.8	156
**Random Forest**	n_est=200, no depth cap	0.9991	0.9991	0.9991	0.9992	0.0001	1.0000	0.0047	43.5	908
**Gradient Boosting**	n_est=100, lr=0.1, max_depth=3	0.9991	0.9991	0.9991	0.9991	0.0001	1.0000	0.0034	2,250.5	2,173
**Neural Network (MLP)**	layers=(128,64), relu, max_iter=500	0.9988	0.9988	0.9988	1	0.0055	1.0000	0.0061	299.1	320
**100k Dataset — 5-fold CV**										
**Logistic Regression**	C=1, lbfgs, multinomial	0.9654	0.9655	0.9655	0.9658	0.0001	0.9978	0.1506	84.5	388
**Random Forest**	n_est=200, no depth cap	0.9994	0.9994	0.9994	0.9994	0.0001	1.0000	0.0026	114.7	1,875
**Gradient Boosting**	n_est=100, lr=0.1, max_depth=3	0.9992	0.9992	0.9992	0.9992	0.0001	1.0000	0.0030	4,964.1	4,294
**Neural Network (MLP)**	layers=(128,64), relu, max_iter=500	0.9993	0.9993	0.9993	0.9991	0.0001	1.0000	0.0023	341.9	604

**Table 9.** Wilcoxon Signed-Rank Significance Tests vs Random Forest.

**Comparison**	**W statistic**	** p-value**	**Δ Mean F1**	**Result**
**50k — 5-fold Wilcoxon (insufficient power at k = 5)**				
RF vs Logistic Regression	0	0.06	0.03	Not significant
RF vs Gradient Boosting	1	0.13	0	Not significant
RF vs Neural Network (MLP)	0	0.06	0	Not significant
**100k — 5-fold Wilcoxon (insufficient power at k = 5)**				
RF vs Logistic Regression	0	0.06	0.03	Not significant
RF vs Gradient Boosting	0	0.06	0	Not significant
RF vs Neural Network (MLP)	0	0.06	0	Not significant
**100k — 10-fold Wilcoxon (for significance testing)**				
RF vs Logistic Regression	0	0.0020*	0.03	* Significant (p < 0.05)
RF vs Gradient Boosting	0	0.0020*	0	* Significant (p < 0.05) — practical Δ negligible
RF vs Neural Network (MLP)	0	0.0020*	0	* Significant (p < 0.05) — practical Δ negligible

5-fold tests are underpowered (k = 5 insufficient for Wilcoxon). 10-fold results are the recommended significance reference. GB excluded from 50k 10-fold due to prohibitive runtime (~4,267s per fold). Practical delta for RF vs GB and RF vs MLP is negligible (Δ < 0.001) at both scales regardless of p-value.

The uncalibrated Random Forest demonstrated exceptional probability calibration (mean ECE = 0.000276, mean Brier score = 0.000143 across ten OvR binary problems), substantially lower than typical values reported in network intrusion detection literature (0.02–0.10). Post-hoc Platt scaling reduced mean ECE to 0.000113, however visual inspection of the reliability diagram confirmed that the uncalibrated model provides more stable probability estimates across the prediction range. This behavior is consistent with known limitations of sigmoid calibration when applied to already well-calibrated models. ^35^ Accordingly, uncalibrated RF probabilities are reported as the primary result, with calibration diagnostics provided in Table 10 and Figure 2 for completeness.

**Table 10.** Calibration diagnostics for the Random Forest classifier (grouped PCA features, 27 components). Post-hoc calibration fitted on the held-out validation set (n = 52,500 at 50k; n = 105,000 at 100k) using cv=‘prefit’. All metrics evaluated on the held-out test set. ECE and Brier scores expressed as integers ×10 ^−6^ for readability (e.g. 276 = 0.000276).

**Method**	**Dataset**	**ECE** **(×10 ^−6^)**	** AUROC-W** **(4 d.p.)**	** Log-Loss** **(4 d.p.)**	**Brier Score (×10 ^−6^)**	**ECE improvement vs uncalibrated**
Uncalibrated RF	50k	276	0.9999	0.0047	143	—
Uncalibrated RF	100k	202	1.0000	0.0026	97	—
Isotonic calibration	50k	98	0.9999	0.0111	132	−65%
Isotonic calibration	100k	51	1.0000	0.0037	86	−75%
Platt (sigmoid) scaling	50k	113	0.9999	0.0049	147	−59%
Platt (sigmoid) scaling	100k	73	1.0000	0.0031	97	−64%

ECE = Expected Calibration Error (10 equal-width bins, averaged across OvR binary problems). Brier = mean Brier score across OvR binary problems.

**Table 11.** Per-class AUROC (OvR) and Brier scores for uncalibrated and isotonic-calibrated Random Forest at both dataset scales. All per-class AUROC values ≥0.9996 at 50k and ≥0.9999 at 100k, with every class improving or holding as dataset size doubled.

**Class**	**50k AUROC**	**50k AUROC (cal.)**	**100k AUROC**	**100k AUROC (cal.)**	** Brier 50k/100k**
BENIGN	1.0000	1.0000	1.0000	1.0000	0.0001/0.0001
DrDoS_LDAP	1.0000	0.9999	1.0000	1.0000	0.0001/0.0000
DrDoS_MSSQL	1.0000	0.9999	1.0000	1.0000	0.0002/0.0001
DrDoS_NTP	1.0000	0.9996	1.0000	0.9999	0.0001/0.0001
DrDoS_NetBIOS	1.0000	0.9999	1.0000	1.0000	0.0001/0.0001
DrDoS_SNMP	1.0000	1.0000	1.0000	1.0000	0.0001/0.0001
DrDoS_UDP	1.0000	0.9999	1.0000	1.0000	0.0002/0.0001
Syn	0.9999	0.9996	1.0000	0.9999	0.0002/0.0001
TFTP	0.9999	0.9999	1.0000	1.0000	0.0001/0.0001
UDP-lag	1.0000	0.9998	1.0000	0.9999	0.0003/0.0002

“cal.” = isotonic calibrated. Brier scores reported for uncalibrated model.

A notable structural finding emerged from comparing results across dataset scales. The grouped PCA pipeline showed an identical component structure at both the 50k and 100k scales; 27 components across six category groups with variance retention varying by no more than 0.60% across any category between the two scales. In contrast, global PCA changed from 22 components at 50k to 21 at 100k, reflecting sensitivity to sampling variation in the overall covariance structure when all 45 features are treated as a single undifferentiated pool ( Table 5). This structural invariance of the grouped approach is a practically important property for operational deployment: retraining on larger datasets does not alter the dimensionality of the feature space passed to downstream classifiers, removing a potential source of pipeline incompatibility in production systems. Furthermore, classification performance improved consistently with scale across all metrics moving weighted F1 from 0.9991 to 0.9994, log loss halving from 0.0047 to 0.0026, and mean ECE improving from 0.000276 to 0.000202, that confirms that both accuracy and probability calibration quality scale favourably with data volume. The grouped PCA configuration also maintained the fastest inference time at both scales (789 ms at 50k, 1,575 ms at 100k), with the inference advantage over raw features growing from 20% to 22% as dataset size doubled, demonstrating that the dimensionality reduction benefit increases with scale ( Table 8).

**Figure 7.**
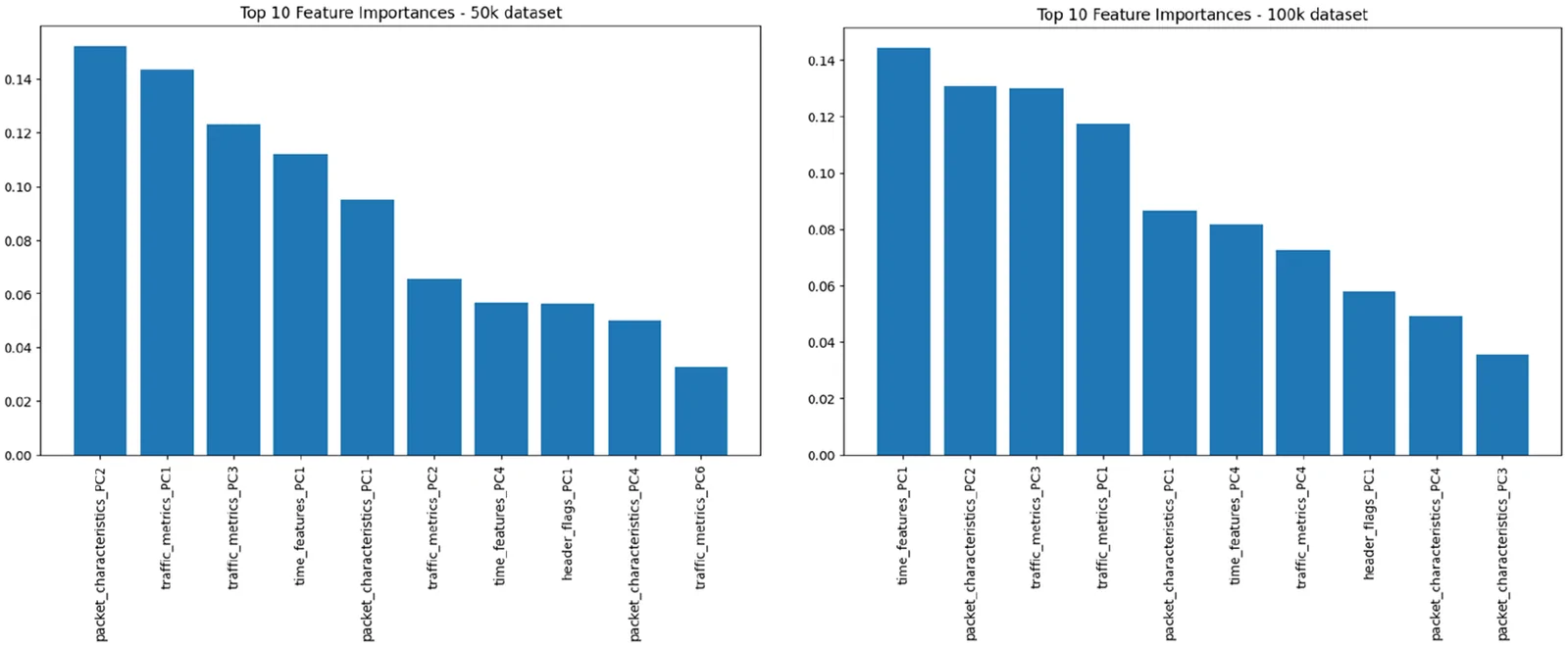
Top 10 features for each of the datasets (50k and 100k).

Of the multiple features involved in the efficiency of the model, a list of the important features responsible for this was analyzed. It was observed that most of the newly generated features were included within the top 10 features based on their importance ( Figure 7 and Table 6), which were observed in both the 50k and 100k datasets. The PCA values that represent the newly generated features, which in turn represent the multiple features categorized under them, have been shown to contribute to the success of the model’s performance.

## 6. Conclusion

By combining feature engineering, dimensionality reduction using PCA, and machine learning for the classification of benign from attack traffic in the CICDDoS2019 dataset, the following conclusions can be drawn from this study. The pivotal concept of feature engineering, capable of capturing the domain-specific nuances of the features within the network traffic, has been a key feature in this efficient classification. In addition to this engineering and processing step, the application of PCA to these derived dataset categories and adding them to form the new dataset led to a significant reduction in the computational burden. A pipeline in which all transformers are fitted exclusively on the training partition was implemented and validated. This pipeline achieved a weighted F1-score of 0.9991 (50k) and 0.9994 (100k), a weighted AUROC of 1.0000 at both scales, log loss of 0.0047 (50k) and 0.0026 (100k), and a mean ECE of 0.000276 (50k) and 0.000202 (100k) on the test sets.

A notable structural finding emerged from comparing the two dataset scales: the grouped PCA pipeline produced an identical component structure at both scales, specifically 27 components across six category groups, while global PCA changed from 22 to 21 components, reflecting sensitivity to sampling variation in the overall covariance structure. This structural invariance is a practically important property for operational deployment, where retraining on larger datasets should not alter the dimensionality of the feature space passed to downstream classifiers. Grouped PCA reduced features from 45 to 27 while lowering inference time and maintaining accuracy. Statistical analysis confirmed the strong performance of Random Forest compared to other classifiers. Random Forest was selected as the primary classifier due to its high accuracy and significantly lower training time. The model also demonstrated near-perfect probability calibration across both dataset scales. Overall, the proposed framework provides a reliable, scalable, and computationally efficient approach for DDoS detection in SDN environments.

## 7. Future work and recommendations

This work could be enhanced by combining advanced models using ensemble techniques, which is an upcoming and promising field that can exploit the advantages of multiple models. Class imbalance has been a major concern in datasets, such as this case, when considering real-world scenarios. This could be improved and addressed to increase the performance of the selected models. Finally, the use of time-series models or a combination of approaches using hybrid approaches could help capture the temporal dependencies present within the network flows, which could in turn improve the accuracy of classification. Several other limitations of the current work should be acknowledged. The CICDDoS2019 dataset is a controlled laboratory capture, and the feature distributions are cleaner and more separable than those typically encountered in production networks; the near-perfect AUROC values reflect this characteristic of the benchmark rather than a universal property of the method. Conducting a cross-dataset validation study by training on CICDDoS2019 and evaluating on a separate dataset such as UNSW-NB15 or CICIDS2017 was not conducted, and remains a priority for future work to establish broader generalisability. Additionally, two attack classes (DrDoS_SSDP and DrDoS_DNS) were excluded from the final model which paves way for future work to investigate the impact of their inclusion and assess whether the model generalises when these traffic types are included. The temporal dimension of network traffic is not covered or modelled in the current approach by incorporating sliding-window flow features or graph-based representations of inter-flow relationships which remain to be considerable future directions that could improve detection of multi-point coordinated attacks.

## Ethical considerations

Ethical approval and consent were not required.

## Data Availability

The data supporting this study are freely available at https://www.unb.ca/cic/datasets/ddos-2019.html last accessed at 31.12.2024. Basic identification details such as Name, Email, Organization, Job title, and country need to be provided for the provider’s internal statistical purpose, and entire dataset can be downloaded.
