# Supplementary material for: Applying Principal Component Analysis for Categorized Dimensionality Reduction in DDoS Detection for Software-Defined Networks
Source: F1000Res. 2026 Jul 27;14:743. Originally published 2025 Jul 28. [Version 2] doi: 10.12688/f1000research.163778.2 (PMC13562928; doi:10.12688/f1000research.163778.2)
Supplement: Peer review history

# Reviewer report: Reviewer response for version 2

**DOI:** 10.5256/f1000research.201849.r509928

**Published:** 2026-09-03

**Authors:** C. M. Nalayini Nalayini

**Affiliations:** 1 Velammal Engineering College, Chennai, India

The manuscript is generally clear and well organized. However, the claims about robustness, scalability, and calibration would benefit from better support and comparison with recent studies.

 The overall methodology appears appropriate, but additional checks for possible data leakage, duplicate samples, and the effect of the train-test splitting strategy would strengthen the reliability of the results.

 Some additional methodological details, particularly regarding dataset preparation, data splitting, feature engineering, PCA component selection, and model settings, are needed to make the study easier to reproduce.

 The statistical and calibration results are promising, but the authors should provide more details on the statistical tests, confidence intervals, and procedures used to obtain the reported calibration measures.

 The conclusions are generally supported by the results, although claims about real-world scalability and adaptability should be stated more cautiously, with a clearer discussion of the limitations of the CICDDoS2019 dataset.

Is the work clearly and accurately presented and does it cite the current literature?

Partly

If applicable, is the statistical analysis and its interpretation appropriate?

Yes

Are all the source data underlying the results available to ensure full reproducibility?

Yes

Is the study design appropriate and is the work technically sound?

Yes

Are the conclusions drawn adequately supported by the results?

Yes

Are sufficient details of methods and analysis provided to allow replication by others?

Yes

Reviewer Expertise:

NETWORK SECURITY MACHINE LEARNING BLOCKCHAIN TECHNOLOGIES

I confirm that I have read this submission and believe that I have an appropriate level of expertise to confirm that it is of an acceptable scientific standard.

# Reviewer report: Reviewer response for version 1

**DOI:** 10.5256/f1000research.180178.r471423

**Published:** 2026-05-06

**Authors:** Hambali Moshood Abiola

**Affiliations:** 1 Federal University Wukari, Wukari, Taraba, Nigeria

This paper addresses a meaningful problem, namely, improving multi-class DDoS detection through domain-driven feature engineering and grouped PCA, and it has several real strengths. The use of a public benchmark, the decision to treat the task as multi-class rather than only binary, the inclusion of classwise metrics, and the effort to articulate a feature engineering rationale all make the study worth serious consideration. The paper also already attracted an open peer review comment that, while generally positive, explicitly asked for stronger statistical justification of design choices and baseline comparisons. 

 At the same time, the manuscript in its current form is not yet methodologically or evidentially strong enough to support its main claims. The most serious problems are internal reporting inconsistencies, especially the mismatch between the abstract’s “50,000” and “100,000” datasets with “eight different class[es]” and the later results table that reports nine class rows, supports of 15,000 or 30,000 per class, and totals of 135,000 and 270,000. In addition, the workflow, as written, places scaling and PCA before the train-test split, which would create leakage if implemented literally. The paper also claims comparisons against global PCA, reduced run times, and consistent results across several additional models, yet it does not provide the corresponding baseline tables, runtime measurements, or model-by-model results. The references contain at least one duplicate and one miscitation, and one cited 2019 paper is inaccurately described as a systematic review. 

 My overall judgment is major revision. The core idea is plausible, but the paper needs a leakage safe reanalysis, corrected data accounting, full ablation baselines, stronger statistical reporting, much clearer reproducibility material, and a more restrained novelty claim. Relative to recent work, the manuscript’s distinct contribution is not PCA itself or feature engineering itself, both of which are already well represented in recent DDoS literature, but the narrower idea of applying PCA separately within domain-defined feature groups after engineered aggregation. That is an incremental contribution, not yet a clearly demonstrated advance. 

 Claimed Findings Versus Evidentiary Standard

 Several of the paper’s most important claims are either under documented or internally inconsistent. These are not minor stylistic matters. They directly affect whether the reported score can be trusted as an estimate of real generalization. 

 Claimed result or design choice    What the manuscript actually shows    Why this is not yet sufficient    What should be added

**“50,000” and “100,000” datasets with “eight different class[es]”  **  The abstract states those counts, but Table 3 reports nine class rows, supports of 15,000 or 30,000 per class, and totals of 135,000 and 270,000.    The arithmetic does not reconcile unless 50k and 100k mean something other than total rows, which is never stated.    Exact total N, exact per class N, train and validation and test counts that sum correctly, and a precise definition of what 50k and 100k refer to.

**Preprocessing before splitting**    The workflow and methods text place scaling and PCA before the 70/30 split.    If implemented as written, this leaks information from the test set into StandardScaler and PCA, inflating performance.    A leakage safe pipeline with train only fitting of every transformer, plus rerun results.

**Comparison against global PCA  **  The introduction says performance was compared against global PCA and significantly improved.    No numerical baseline table for global PCA appears in the results section.    An ablation table showing raw features, engineered only, global PCA, and grouped PCA under the same classifier and same split.

**Evaluation metrics include ROC AUC and log loss  **  Methods list ROC AUC and log loss, but the main result table reports precision, recall, F1, accuracy, and cross validated F1 only.    Important probabilistic and ranking evidence is missing. For multi class problems, the exact AUROC formulation also matters.    Macro and weighted one versus rest AUROC, log loss, and preferably calibration diagnostics, each with uncertainty.

**Multiple algorithms were tested consistently **   The text says Gradient Boost, Logistic Regression, SVM, and a neural network were tested, but the paper does not show a proper comparison table with their metrics and settings.    Without the actual numbers, this cannot support the claim of broad robustness.    A model comparison table with all hyperparameters, all primary metrics, runtime, and paired significance testing versus the main baseline.

**PCA caused “no information loss” and reduced runtime  **  The manuscript also says only 95 percent variance was retained and gives no runtime table.    Retaining 95 percent variance is not the same as zero information loss, and runtime reduction is unsubstantiated without timing data.    Replace the wording with “retained most variance,” provide explained variance tables per category, and report fit and inference time on stated hardware.

Is the work clearly and accurately presented and does it cite the current literature?

Yes

If applicable, is the statistical analysis and its interpretation appropriate?

Not applicable

Are all the source data underlying the results available to ensure full reproducibility?

Yes

Is the study design appropriate and is the work technically sound?

Partly

Are the conclusions drawn adequately supported by the results?

Yes

Are sufficient details of methods and analysis provided to allow replication by others?

Partly

Reviewer Expertise:

Machine Learning and Information Security

I confirm that I have read this submission and believe that I have an appropriate level of expertise to confirm that it is of an acceptable scientific standard, however I have significant reservations, as outlined above.

# Reviewer report: Reviewer response for version 1

**DOI:** 10.5256/f1000research.180178.r440059

**Published:** 2026-01-12

**Authors:** Dr.V.Latha Jothi, Gokulraj S, Russia S

**Affiliations:** 1 Velalar College of Engineering and Technology, Erode, India; 2 Dr NGP Institute of Technology (Ringgold ID: 267149), Coimbatore, Tamil Nadu, India

The article presents a well-structured and methodical approach to DDoS attack detection in Software-Defined Networks using domain-driven feature engineering and categorized PCA. The methodology is clearly explained and the use of the CICDDoS2019 dataset with appropriate evaluation metrics demonstrates the robustness and scalability of the proposed model. The analysis could be strengthened by providing additional statistical justification for certain design choices, such as parameter selection, PCA variance thresholds and comparative statistical significance testing against baseline models.

Is the work clearly and accurately presented and does it cite the current literature?

Yes

If applicable, is the statistical analysis and its interpretation appropriate?

Partly

Are all the source data underlying the results available to ensure full reproducibility?

Yes

Is the study design appropriate and is the work technically sound?

Yes

Are the conclusions drawn adequately supported by the results?

Yes

Are sufficient details of methods and analysis provided to allow replication by others?

Yes

Reviewer Expertise:

Artificial Intelligence, Machine Learning, Image Processing

We confirm that we have read this submission and believe that we have an appropriate level of expertise to confirm that it is of an acceptable scientific standard.
